# Identification and characterization of the *Fasciola hepatica* sodium- and chloride-dependent taurine transporter

**DOI:** 10.1371/journal.pntd.0006428

**Published:** 2018-04-27

**Authors:** Bulut Hamali, Sandra Pichler, Elisabeth Wischnitzki, Klaus Schicker, Melanie Burger, Marion Holy, Kathrin Jaentsch, Martina Molin, Eva Maria Sehr, Oliver Kudlacek, Michael Freissmuth

**Affiliations:** 1 Institute of Pharmacology, Center of Physiology and Pharmacology, Medical University of Vienna, Vienna, Austria and Gaston H. Glock Research Laboratories for Exploratory Drug Development, Vienna, Austria; 2 Center for Health & Bioresources, AIT Austrian Institute of Technology GmbH, Tulln, Austria; University of Pennsylvania, UNITED STATES

## Abstract

The parasitic liver fluke *Fasciola hepatica* infests mainly ruminants, but it can also cause fasciolosis in people, who ingest the metacercariae encysted on plants. The drug of choice to treat fasciolosis is triclabendazole (TBZ), which has been on the market for several decades. This is also true for the other available drugs. Accordingly, drug-resistant flukes have been emerging at an increasing rate making it desirable to identify alternative drug targets. Here, we focused on the fact that adult *F*. *hepatica* persists in the hostile environment of the bile ducts of infected organisms. A common way to render bile acids less toxic is to conjugate them to taurine (2-aminoethanesulfonic acid). We cloned a transporter from the solute carrier-6 (SLC6) family, which was most closely related to the GABA-transporter-2 of other organisms. When heterologously expressed, this *F*. *hepatica* transporter supported the high-affinity cellular uptake of taurine (K_M_ = 12.0 ± 0.5 μM) but not of GABA. Substrate uptake was dependent on Na^+^- and Cl^-^ (calculated stoichiometry 2:1). Consistent with the low chloride concentration in mammalian bile, the *F*. *hepatica* transporter had a higher apparent affinity for Cl^-^ (EC_50_ = 14±3 mM) than the human taurine transporter (EC_50_ = 55±7 mM). We incubated flukes with unconjugated bile acids in the presence and absence of taurine: taurine promoted survival of flukes; the taurine transporter inhibitor guanidinoethansulfonic acid abolished this protective effect of taurine. Based on these observations, we conclude that the taurine transporter is critical for the survival of liver flukes in the bile. Thus, the taurine transporter represents a candidate drug target.

## Author summary

The liver fluke *F*. *hepatica* imposes an economic burden by infesting domestic ruminants. In addition, the WHO classified human fasciolosis as a disease of vital global public health importance, because several million people worldwide are infested by this trematode. Because it is currently not possible to vaccinate against this parasite, anthelmintic drugs are the treatment of choice for both, animals and people. During the last decades, the proportion of flukes resistant to drugs has steadily increased. *F*. *hepatica* persists in the hostile environment of the host bile duct. Accordingly, the fluke must be endowed with defense mechanisms, which protect it against the toxic actions of bile acids. This working hypothesis was explored by cloning a candidate taurine transporter of *F*. *hepatica*. The experiments verified that the transporter mediated the sodium- and chloride-dependent uptake of taurine. It requires only low concentrations of chloride, which indicates adaptation to the ionic composition of the bile, and it is protective: inhibition of the transporter renders flukes susceptible to killing by bile acids. By showing that the taurine transporter represents an Achilles heel of *F*. *hepatica*, these observations point to a new anthelmintic therapeutic strategy.

## Introduction

Liver flukes of the genus *Fasciola* are parasitic trematodes, which infest mammals all over the world. The two most prominent representatives of the genus *Fasciola* are *Fasciola hepatica* and *Fasciola gigantica*, which predominates in the temperate zones and tropical Africa and Asia, respectively [1]. The resulting fasciolosis imposes a substantial economic burden because of the decrease in milk production, weight gain and wool yield and due to sudden deaths of livestock animals [2–5]. People are infested by ingestion of metacercariae encysted on plants or ingestion of water containing metacercariae. This not only can give rise to regional pockets of endemic infections, e.g. on the Bolivian Altiplano [6], but it is also relevant to human health worldwide: conservative estimates indicate that more than 2.5 million people are infested and suffer from various forms of fasciolosis [7]. The ingested metacercariae excyst in the duodenum. The process is triggered by chemical cues including elevated carbon dioxide levels, requires the sequential action of host and parasite proteases [8] and is contingent on the presence of bile acids [9]. The emerging juvenile flukes also depend on their proteases to invade and penetrate the host gut wall [10]. In the peritoneal cavity, the juvenile flukes migrate to the liver—presumably using the curvature of the abdominal wall as a guidance clue [11]. They subsequently burrow through the liver capsule and feed on the tissue for several weeks, until they are mature. The mature flukes invade the bile ducts, which allows for sexual reproduction. By contrast with *F*. *hepatica*, the Chinese liver fluke *Clonorchis sinensis* invades the bile duct within two days after excysting. Finally, *Opisthorchis viverrini*, the Southeast Asian liver fluke, invades the bile ducts via retrograde migration through the coledochus. However, in spite of the differences in their life cycles, *F*. *hepatica*, *C*. *sinensis* and *O*. *viverrini* eventually face the same hostile environment of the bile.

A vaccine against *F*. *hepatica* is desirable, but there are many obstacles which impede the development of an effective active immunization [12]. Accordingly, fasciolosis is treated by anthelmintic chemotherapy. Triclabendazole has been the drug of choice for more than 35 years: triclabendazole is highly active against both adult and juvenile flukes and is well tolerated by the mammalian hosts. Predictably, flukes resistant to triclabendazole have emerged [13]. Resistance is also seen with other anthelmintic compounds which kill *F*. *hepatica*, e.g. albendazole, clorsulon, closantel, oxyclozanide and nitroxynil [13–15]. Most mammals do not mount an effective immune response to liver flukes [12,16]. Hence adult *F*. *hepatica* can survive for many years within the biliary tract: adult worms were retrieved after 11 years from an experimentally infected sheep [17]. A case report from an elderly patient suggests that *F*. *hepatica* can also reach this age in people [18]. Motile flukes have been visualized by radiological imaging techniques in the gallbladder and in the common bile duct of patients [19]. In fact, whole bile and some bile acids such as glycine-conjugated cholic acid and dehydrocholic acid stimulate the mobility of juvenile flukes [20,21]. This indicates that some bile acids provide a chemokinetic or chemotactic signal. However, bile acids, in particular, deoxycholic acid, are also toxic to flukes [21]. Thus, while juvenile and adult *F*. *hepatica* are attracted by bile constituents, protective mechanisms must have evolved, which allow them to survive the toxic effects of the bile, e.g. by conjugating taurine to bile acids. Here we surmised that flukes rely on taurine transport to cope with bile acids. We cloned a candidate taurine transporter, confirmed its biochemical activity and verified that inhibition of taurine transport rendered adult flukes susceptible to killing by bile acids.

## Results

### Primary structure of the *F*. *hepatica* taurine transporter (FhTauT)

Transcripts encoding the orthologue of the human taurine transporter SLC6A6 were identified from a sequence database including mRNA data of adult *F*. *hepatica* [22]. A fragment of about 0.8 kB was identified via the sequence analysis and thereafter extended by amplifying the cDNA ends. An in-frame stop codon is present 5 triplets upstream of the first ATG (S1A Fig), which indicates that the translation start site has been correctly identified. In addition, the sequence flanking the first ATG conforms to a canonical Kozak sequence with guanine bases at positions -6, -3 and +4. The 3' end of the amplified fragment also comprised the polyadenylation signal (starting with A2746, S1A Fig) and the initial portion of the poly-A tail indicating that the amplified fragment covered the entire mRNA. The open reading frame of 1944 bp encodes a protein of 647 amino acids (S1A Fig) corresponding to a relative molecular mass of 71,939 Da. The sequence was deposited at GenBank with the accession number: MG674191 as *F*. *hepatica* taurine transporter (FhTauT). The exon-intron boundaries were identified by comparing the obtained cDNA sequence with the genomic sequence deposited in the database WormBase (http://parasite.wormbase.org/) [23,24]. This analysis led to the prediction that the FhTauT was encoded by 14 exons (S1B Fig). We also identified the putative taurine transporter in the *F*. *gigantica* transcriptome [19]: a comparison of the predicted amino acid sequences revealed that the two transporters differed at 12 of 647 positions resulting in 98% identity (S2 Fig).

**Fig 1:**
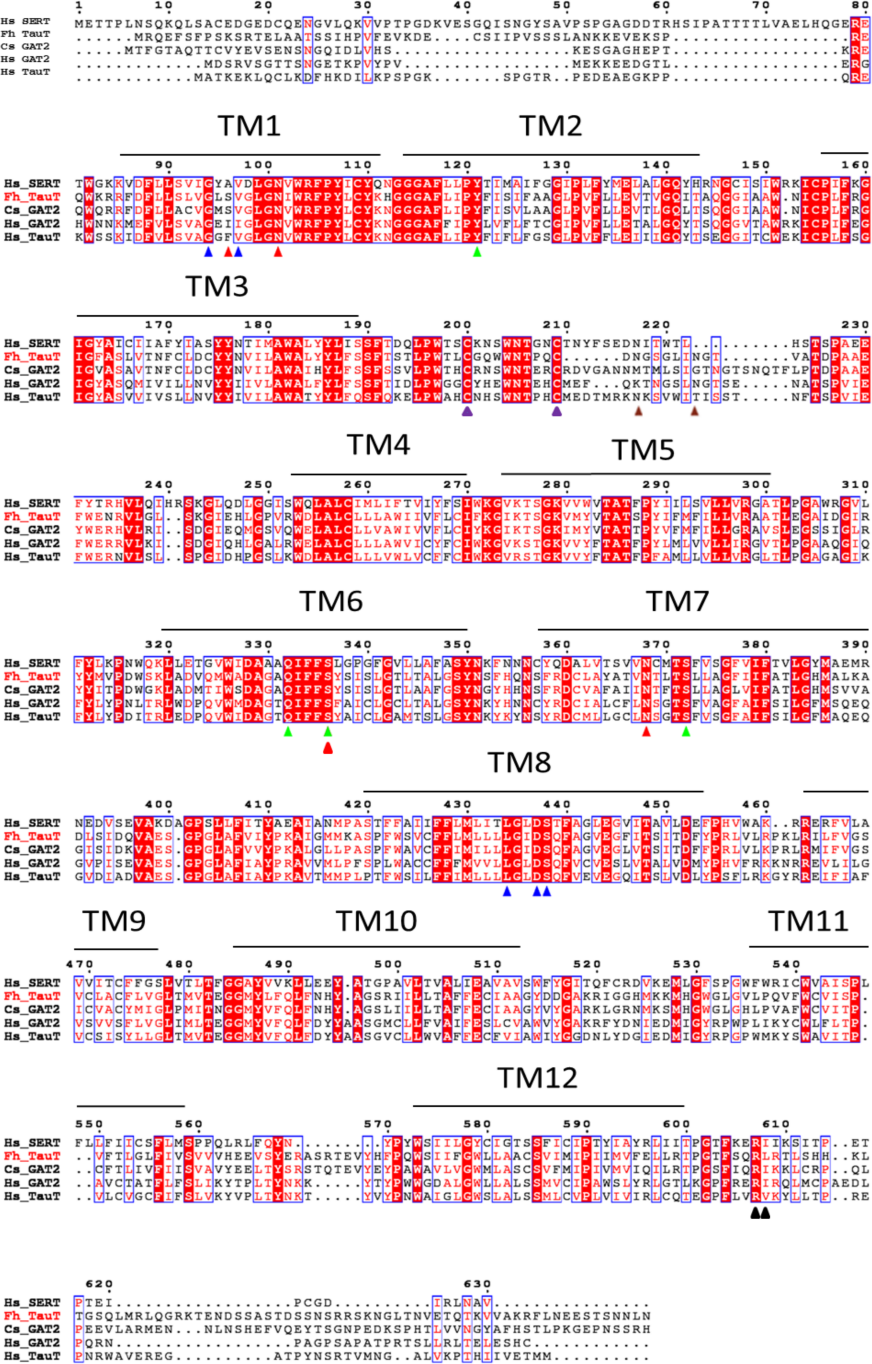
Amino acid alignment of the *F*. *hepatica* taurine transporter (Fh TauT) with the human serotonin transporter (Hs SERT), the GABA-transporter-2 of *C*. *sinensis* (Cs GAT2), the human GABA-transporter-2 (Hs GAT2) and the human taurine transporter (Hs TauT). Amino acids are represented in their single-letter code. Invariant sequences are denoted by white letters boxed in red, sequences with conservative substitutions are denoted by red letters. The red and blue arrow heads mark amino acids contributing to the first and second Na^+^ binding sites, respectively, in the crystal structure of Hs SERT. The green arrow heads highlight residues involved in the coordination of Cl^-^ in SERT. The cysteine residues in the second extracellular loop, which form a putative disulfide bridge, are indicated by magenta arrow heads. The brown arrow heads indicate candidate sites for N-linked glycosylation predicted by NetNGlyc 1.0 (http://www.cbs.dtu.dk/services/NetNGlyc/).

We aligned the amino acid sequence of the putative taurine transporter of *F*. *hepatica* to three sequences of human SLC6 family members—i.e. the serotonin transporter (SERT/SLC6A4), the GABA transporter-2 (GAT2/SLC6A13), the taurine transporter (TauT/SLC6A6)—and the putative GABA-transporter-2 of the trematode *C*. *sinensis*, another liver fluke. It is evident from Fig 1 that there is extensive sequence conservation within the hydrophobic core, which is comprised of the twelve transmembrane (TM) segments. The structure of several SLC6 transporters is understood in atomic detail. In the crystal structure of SERT [25], the Na^+^ binding site is comprised of A^96^ and N^101^ in TM1, S^336^ in TM6 and N^368^ in TM7 (marked by red arrow heads in Fig 1): these residues (i.e. N^73^, S^303^ and N^335^) are conserved in the *F*. *hepatica* taurine transporter with the exception of A^96^ in human SERT, which is replaced by a serine (S^68^). Similarly, the residues forming the binding site of the second sodium ion in human SERT (G^94^ and V^97^ in TM1, L^434^, D^437^ and S^438^ in TM8 marked by blue arrow heads in Fig 1), are identical in the *F*. *hepatica* taurine transporter (G^66^, V^69^, L^401^, L^434^, S^435^). In SERT, the chloride ion is coordinated by Y^121^ of TM2, Q^332^ and S^336^ of TM6 and S^372^ of TM7 (marked by green arrow heads in Fig 1). These residues are also invariant (Y^93^, Q^299^, Q^303^, S^339^). SLC6 transporters have a long second extracellular loop (EL2), which is stabilized by a disulfide bond. The candidate cysteine residues (C^171^ and C^181^ marked by magenta arrow heads in Fig 1) are also present in the *F*. *hepatica* taurine transporter. SLC6 transporters have a variable number of N-linked glycosylation sites; SERT has a single site (Fig 1), but the dopamine transporter DAT (SLC6A3) has three [26,27]. We identified two asparagine residues which conform to the NXS/T-glycosylation motif in the *F*. *hepatica* taurine transporter (N^183^ and N^189^, marked by brown arrow heads in Fig 1). Finally, with the notable exception of the neutral amino acid transporters B^0^AT3/SLC6A18 and B^0^AT1/SLC6A19 [28], SLC6 transporters harbor a SEC24-binding site in their C-terminus: this RI/RL-motif [29–31] that is also present in the *F*. *hepatica* taurine transporter (R579/I580, black arrow heads in Fig 1). The +2 residue (T^582^) is hydrophilic, which predicts that the *F*. *hepatica* transporter recruits SEC24C for ER-export [31].

### Phylogenetic analysis of the putative *F*. *hepatica* transporter

**Fig 2:**
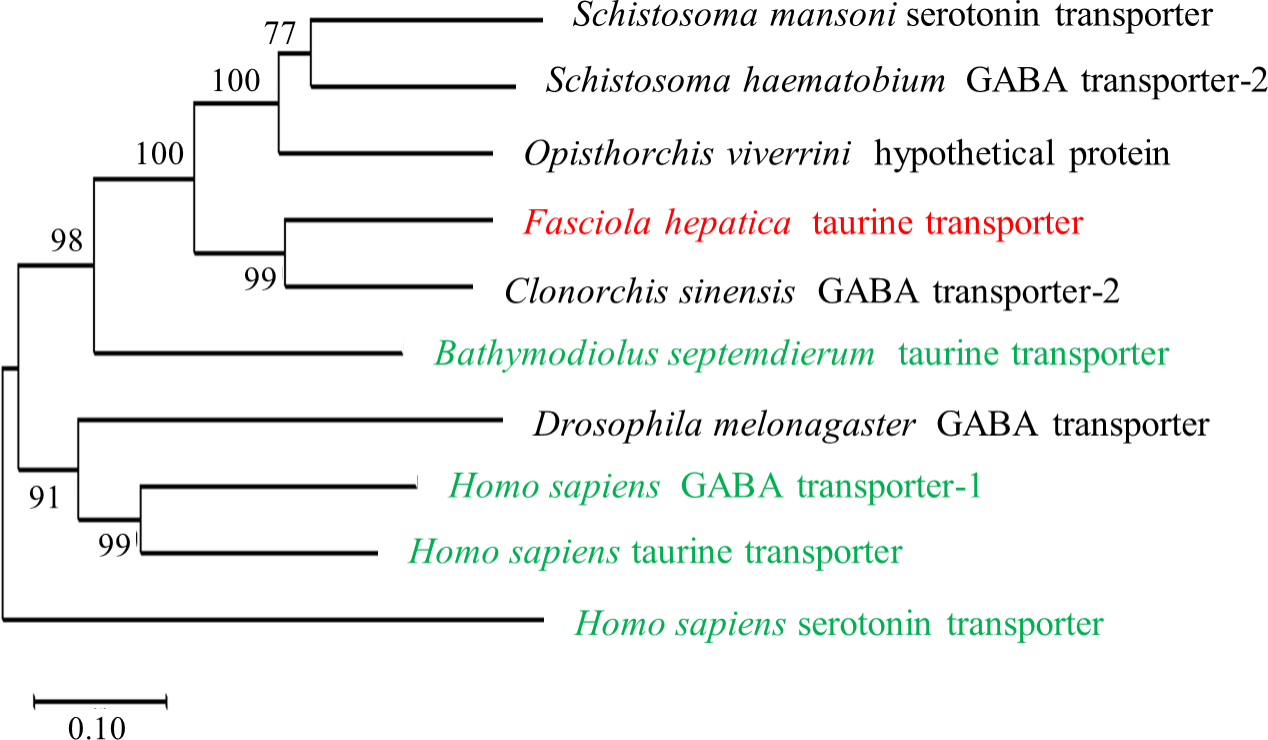
Phylogenetic relation of the *F*. *hepatica* taurine transporter with transporters of other organisms. The neighbor-joining method was used to construct the phylogenetic tree by comparing amino acid sequences of transporters (for accession numbers of sequences see materials and methods) with MEGA7 [34]. The percentage of replicate trees in which the associated taxa clustered together in the bootstrap test (1000 replicates, Poisson correction) are shown next to the branches. The scale bar reflects the likelihood that a change in amino acid occurred on any given branch. The transporters written in green indicate those SLC6 family members, where the substrate uptake has been verified in biochemical experiments and/or by electrophysiological recordings. For transporters written in black, the functional assignment is listed as deposited in the data bases; it is apparently based on sequence homology.

We used the basic local alignment search tool (BLAST) to identify the closest relatives of the putative *F*. *hepatica* taurine transporter. As expected, this search retrieved SLC6 transporters from other digenean trematodes—i.e., *Clonorchis sinensis*, *Opisthorchis viverrini*, *Schistosoma mansoni*, *Schistosoma haematobium*—and taurine or GABA transporters from other species (e.g., the deep-sea mollusc *Bathymodiolus septemdierum*, *Drosophila melanogaster* and *Homo sapiens)*. A phylogenetic tree was constructed based on the identified sequences (Fig 2). The GABA-transporter 2 from *C*. *sinensis* and the *F*. *hepatica* taurine transporter showed the highest pairwise identity of 65%. The evolutionary relation of digenean trematodes has been clarified based on the comparison of 12 proteins encoded by their mitochondrial genomes [32]: this shows that Opisthorchiidae and Fasciolidae (and Paragonimidae) are closely related, while flukes of the Schisostomatidae family are assigned to a separate branch. The evolutionary tree depicted in Fig 2 only recapitulated this relation in part, because the (hypothetical) SLC6 transporter form *Opisthorchis viverrini* was assigned to the same branch as the *Schistosoma* transporters for GABA and serotonin. However, it is worth pointing out that the biochemical activity of the majority of these transporters has only been inferred: in fact, of the non-human transporters shown in Fig 2, it is only clear that the taurine transporter of *B*. *septemdierum* mediates the uptake of its eponymous substrate [33]. It is therefore questionable that the assigned names on the phylogenetic tree are correct: it is, for instance, difficult to understand, why the serotonin transporter of *S*. *mansoni* should be more closely related to the *S*. *mansoni* GABA-transporter-2 than to the human serotonin transporter (Fig 2). In fact, when the sequences of Platyhelminthes deposited in the WormBase database were subjected to a homology search, several close relatives to the putative FhTauT were found. The corresponding alignment is shown in S3 Fig. It is evident from this alignment that the Digenean transporters (marked by the gray area in S3 Fig) are more closely related to each other than those of the other Platyhelminthes. We stress that there are still several uncertainties in this comparison because it is not clear whether all these transporters are taurine transporters. For *Opisthorchis viverrini* we managed to identify a second transporter highly homologous to the taurine transporter of *F*. *hepatica*. In contrast to the originally identified transporter (underlined *Opisthorchis viverrini* in S3 Fig), the second transporter clusters as would be expected to the transporter of *C*. *sinensis* and not the the transporters of the Schistosoma family. We therefore conclude, although functional data are missing, that this second transporter is the transporter for taurine. For all other platyhelminthic transporters described in Fig 2, we could not find other transporters aligning better to the taurine transporter of *F*. *hepatica*.

### Heterologous expression of the *F*. *hepatica* taurine transporter in HEK293 cells

**Fig 3:**
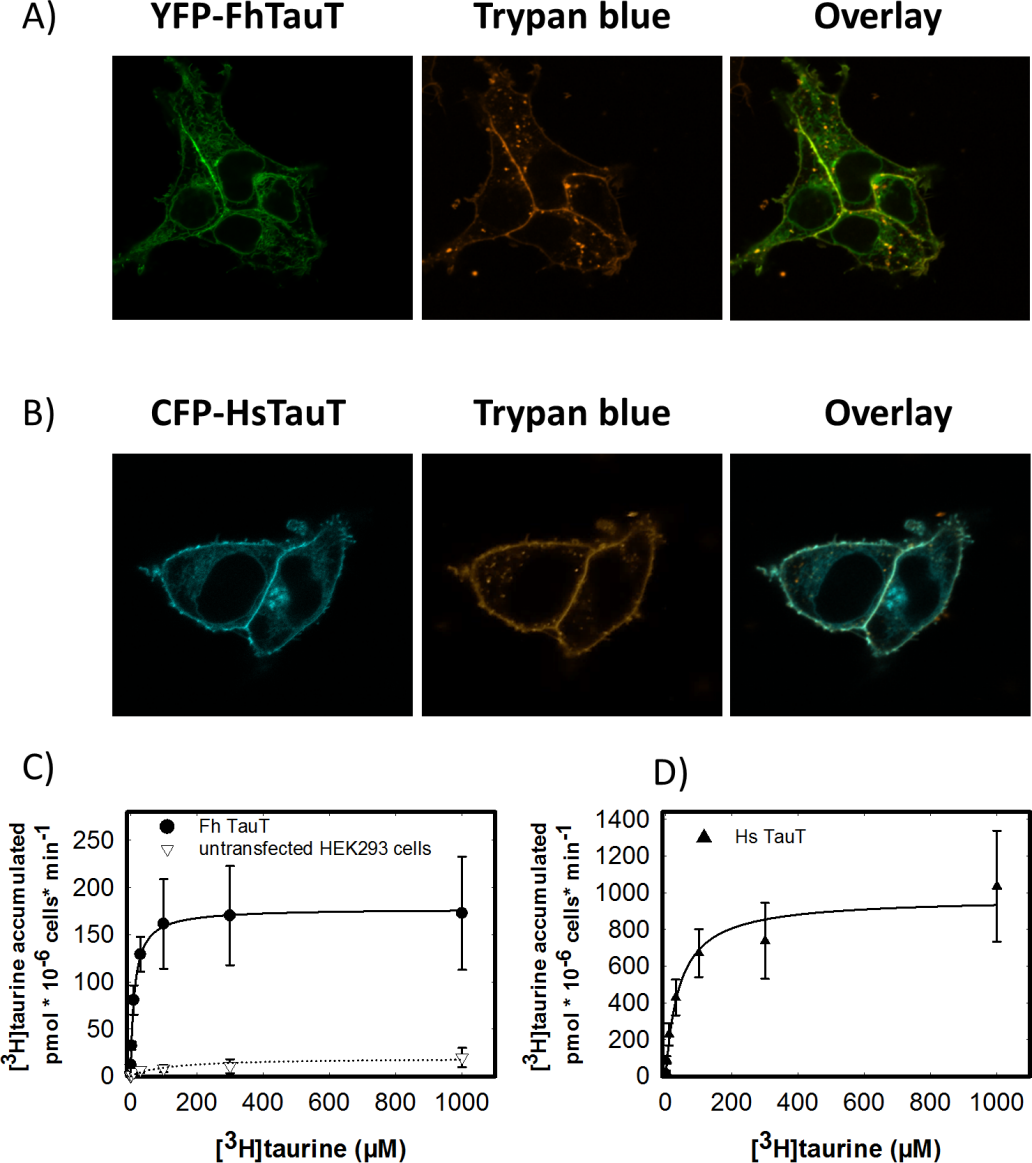
**Cellular localization of (A, B) and substrate uptake (C, D) by the *F*. *hepatica* (A, C) and the human taurine transporter (B, D) after heterologous expression in HEK293 cells.** HEK293 cells were stably transfected with plasmids encoding fluorescently tagged versions of the *F*. *hepatica* (YFP-FhTauT, panel A) and the human (CFP-HsTauT, panel B) taurine transporter. The cellular distribution of the tagged transporters was visualized by confocal microscopy (left-hand images). The cell surface was delineated by staining with trypan blue (images in the middle). The captured confocal images were overlaid (right-hand images). *C & D*: HEK 293 cells (1*10^5^ cells/well) expressing the YFP-tagged *F*. *hepatica* taurine transporter (circles in C) and the YFP-tagged human taurine transporter (circles in D) or untransfected HEK 293 cells (open triangles in C) were incubated for 10 min in the presence of the indicated concentration of [^3^H]taurine. The accumulated radioactivity was determined as described under *Materials and Methods*. Data are means ± S.D. of at least three independent experiments, which were carried out in duplicate. The solid lines were drawn by fitting the data to the equation of a rectangular hyperbola.

The sequence comparison in Fig 1 and the evolutionary tree in Fig 2 highlight the close relation between taurine and GABA transporters. The substrate specificity of the putative *F*. *hepatica* transporter was verified by heterologous expression in HEK293 cells. The human taurine transporter was used as a reference. SLC6 transporters can be tagged with fluorescent proteins on their N-terminus without affecting their activity [35] and their trafficking through the secretory pathway [36–38]. Accordingly, we introduced the cDNA encoding the *F*. *hepatica* and the human taurine transporter into mammalian expression vectors, which fused the coding sequence of CFP and YFP, respectively, in frame to the N-terminus of the transporters. This allowed for visualizing the cellular distribution of the transporters by confocal microscopy after heterologous expression in HEK293 cells: the plasma membrane was delineated by staining with trypan blue. A larger fraction of the *F*. *hepatica* taurine transporter (Fig 3A) than of the human transporter (Fig 3B) seems to be retained within the cell. However, both transporters reached the cell surface. Accordingly, cells expressing the *F*. *hepatica* and the human taurine transporter and untransfected control cells were incubated in the presence of [^3^H]taurine and of [^3^H]GABA to determine the substrate specificity. We failed to detect any uptake of [^3^H]GABA (S4 Fig). In contrast, cells expressing the *F*. *hepatica* taurine transporter accumulated substantially (i.e., about 10-fold) higher amounts of [^3^H]taurine (closed circles in Fig 3C) than untransfected control cells (open triangles in Fig 3C). Transport by both the *F*. *hepatica* (Fig 3C) and the human transporter (Fig 3D) was adequately described by a rectangular hyperbola. The K_M_ of the human transporter was 41.0 ± 10.4 μM, which is in excellent agreement with that originally reported in a similar uptake assay (43±6 μM) [39]. The apparent affinity of the *F*. *hepatica* transporter for taurine (K_M_ = 12.0 ± 0.5 μM) was higher than that of the human transporter. In contrast, the maximum velocity of uptake was lower in cells expressing the *F*. *hepatica* taurine transporter (178 ± 2 pmol*10^−6^ cells*min^-1^) than in cells expressing the human taurine transporter (971.0 ± 60.3 pmol*10^−6^ cells*min^-1^, Fig 3D). This can be accounted for—at least in part—by the lower surface expression of the *F*. *hepatica* taurine transporter (Fig 3A).

**Fig 4:**
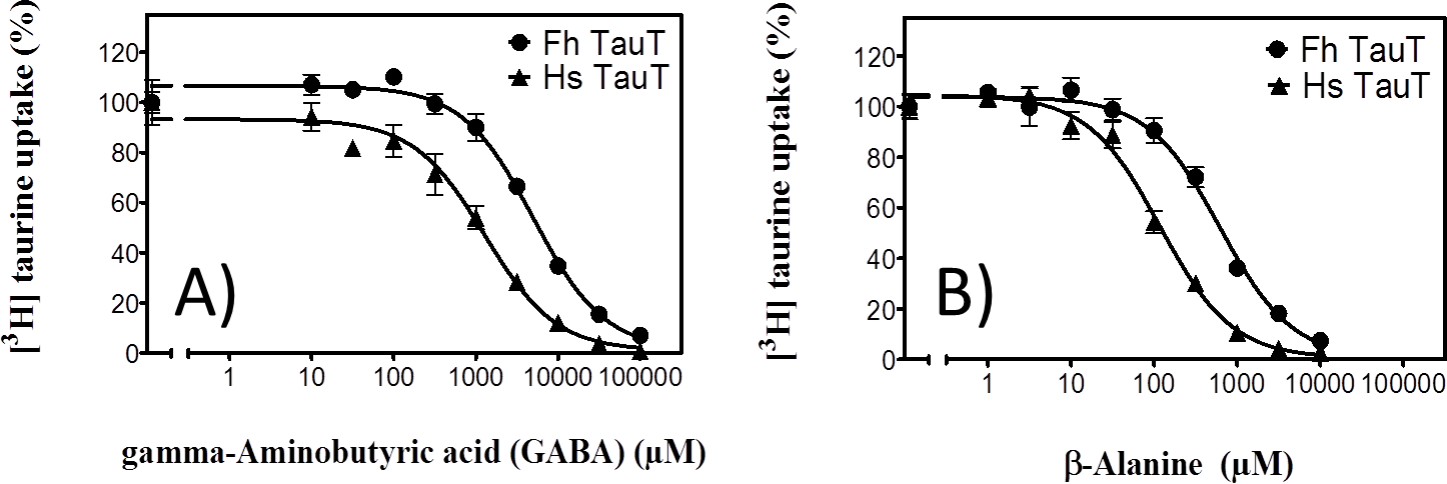
**GABA- (A) and β-alanine-induced (B) inhibition of [**^**3**^**H]taurine uptake by the *F*. *hepatica* and the human taurine transporter after heterologous expression in HEK293 cells.** Stably transfected HEK 293 cells (1*10^5^ cells/well) expressing the YFP-tagged F. hepatica taurine transporter (circles, YFP-FhTauT) or the YFP-tagged human taurine transporter (triangles, CFP-HsTauT) were incubated for 10 min in the presence of 0.1 μM [3H]taurine and the indicated concentration of GABA (A) and β-alanine (B). The accumulated radioactivity was determined as described under Materials and Methods. Uptake observed in the absence of any inhibitor was set at 100% to normalize for interassay variability. These 100% control values were 0.89 ± 0.05 and 1.102 ± 0.142 pmol.min^-1^.10^−6^ cells for the *F*. *hepatica* and the human transporter, respectively. Data are means ± S.D. from three independent experiments. The solid lines were drawn by fitting the data to the equation for a monophasic inhibition curve.

We also examined the ability of unlabelled GABA to inhibit uptake of [^3^H]taurine to estimate the affinity of the *F*. *hepatica* transporter for GABA: High concentrations of GABA were required to suppress taurine uptake by the *F*. *hepatica* transporter (Fig 4A). From the monophasic inhibition curves, we calculated IC_50_-values of 5.6 ± 0.9 mM. As a reference, we assessed the affinity of the human taurine transporter for GABA in parallel (triangles in Fig 4A): GABA was more potent in inhibiting the human than the fluke transporter; the estimated IC_50_ of GABA (1.6 ± 0.5 mM) is in agreement with the reported K_M_ of 1.46 mM [40]. β-Alanine was originally proposed as the alternative substrate of the taurine transporter [41]. Accordingly, we also compared the apparent affinity of the human and the *F*. *hepatica* transporter for β-alanine (Fig 4B): β-alanine was more potent in inhibiting [^3^H]taurine uptake by the human transporter (IC_50_ = 132 ± 54 μM) than by the fluke transporter (IC_50_ = 713 ± 260 μM). The affinity estimate for the human transporter was in line with the original observation that 100 μM β-alanine inhibited uptake by about 50% [39].

**Fig 5:**
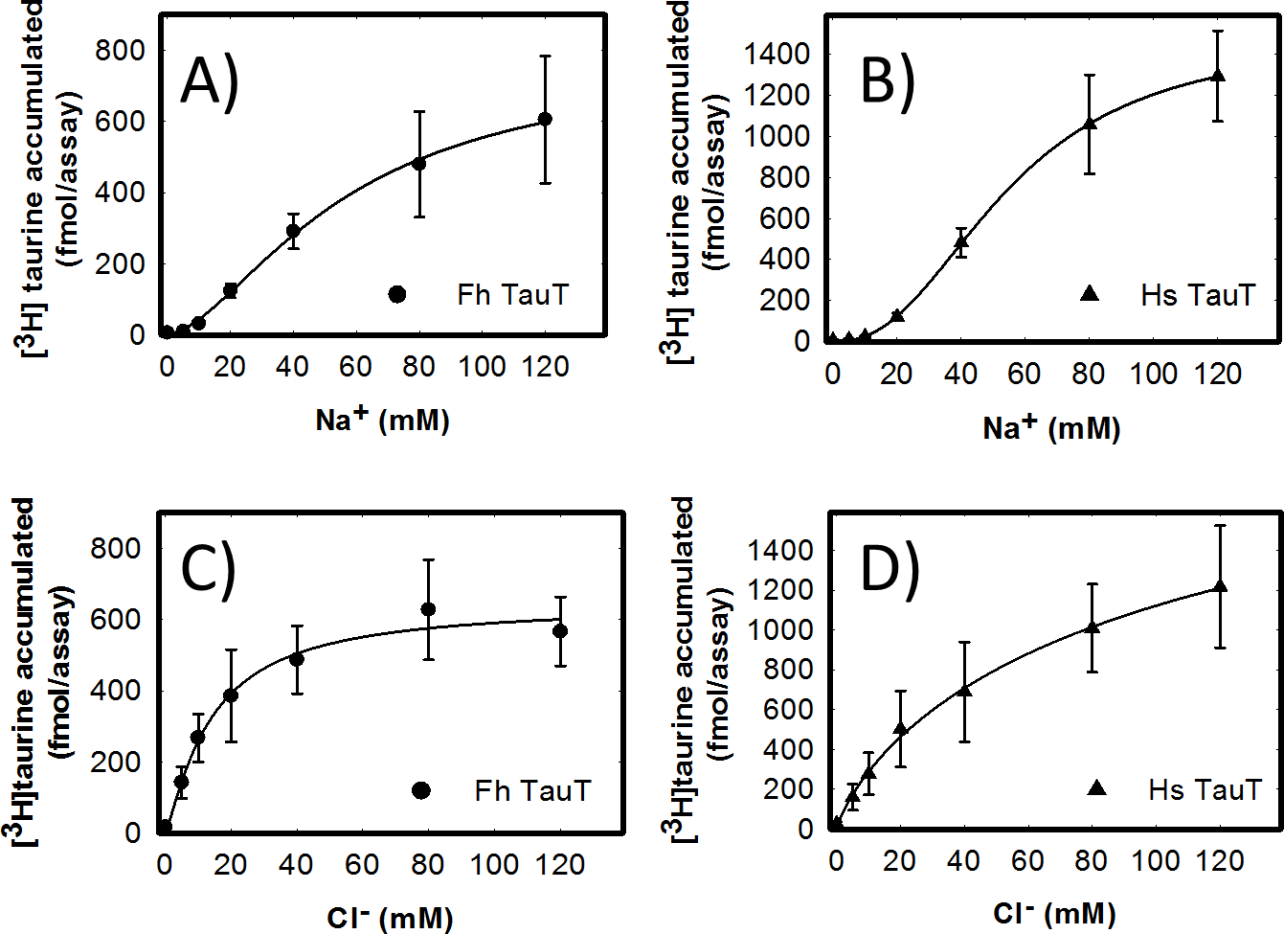
**Sodium- (A, B) and chloride-dependence (C, D) of [**^**3**^**H]taurine uptake by the *F*. *hepatica* and the human taurine transporter.** HEK293 cells (1*10^5^ cells/well) stably expressing the *F*. *hepatica* (A, B) or the human taurine transporter (C, D) were incubated for 10 min in the presence of 0.1 μM [^3^H]taurine and of the indicated concentrations of sodium (A, B) and chloride (C, D). The ionic strength was kept constant by replacing sodium with choline (A, B) or by substituting chloride with acetate (C, D). Data represent means ± S.D. from three independent experiments carried out in duplicate. The solid lines were drawn by fitting the data to the Hill equation.

Substrate transport by SLC6 family members requires the co-transport of two or three Na^+^ ions. These provide the driving force for intracellular accumulation of the substrate. In addition, most SLC6 transporters are dependent on chloride, although the chloride gradient is immaterial, because the transporter completes the transport cycle in a chloride-bound return step [42]. We compared the ionic requirement of the *F*. *hepatica* (Fig 5A & 5C) and the human transporter (Fig 5B & 5D) by isoosmotic replacement of sodium with choline (Fig 5A & 5B) and of chloride with acetate (Fig 5C & 5D). Both transporters required similar amounts of Na^+^ for half-maximum stimulation of transport (EC_50_ = 51.4 ± 6.0 mM and 56.5 ± 7.1 mM for the *F*. *hepatica* and the human transporter, respectively). Similarly, the sodium saturation curves were sigmoidal with Hill coefficients of 1.6±0.1 and 2.3±0.1 for the *F*. *hepatica* and the human transporter, respectively. In contrast, the chloride saturation curves were hyperbolic (Fig 5C & 5D). It is also evident from the comparison of Fig 5C and 5D that substrate uptake by the *F*. *hepatica* transporter required substantially lower concentrations of chloride (EC_50_ = 13.9±3.1 mM) than the human transporter (54.8±6.9 mM).

**Fig 6:**
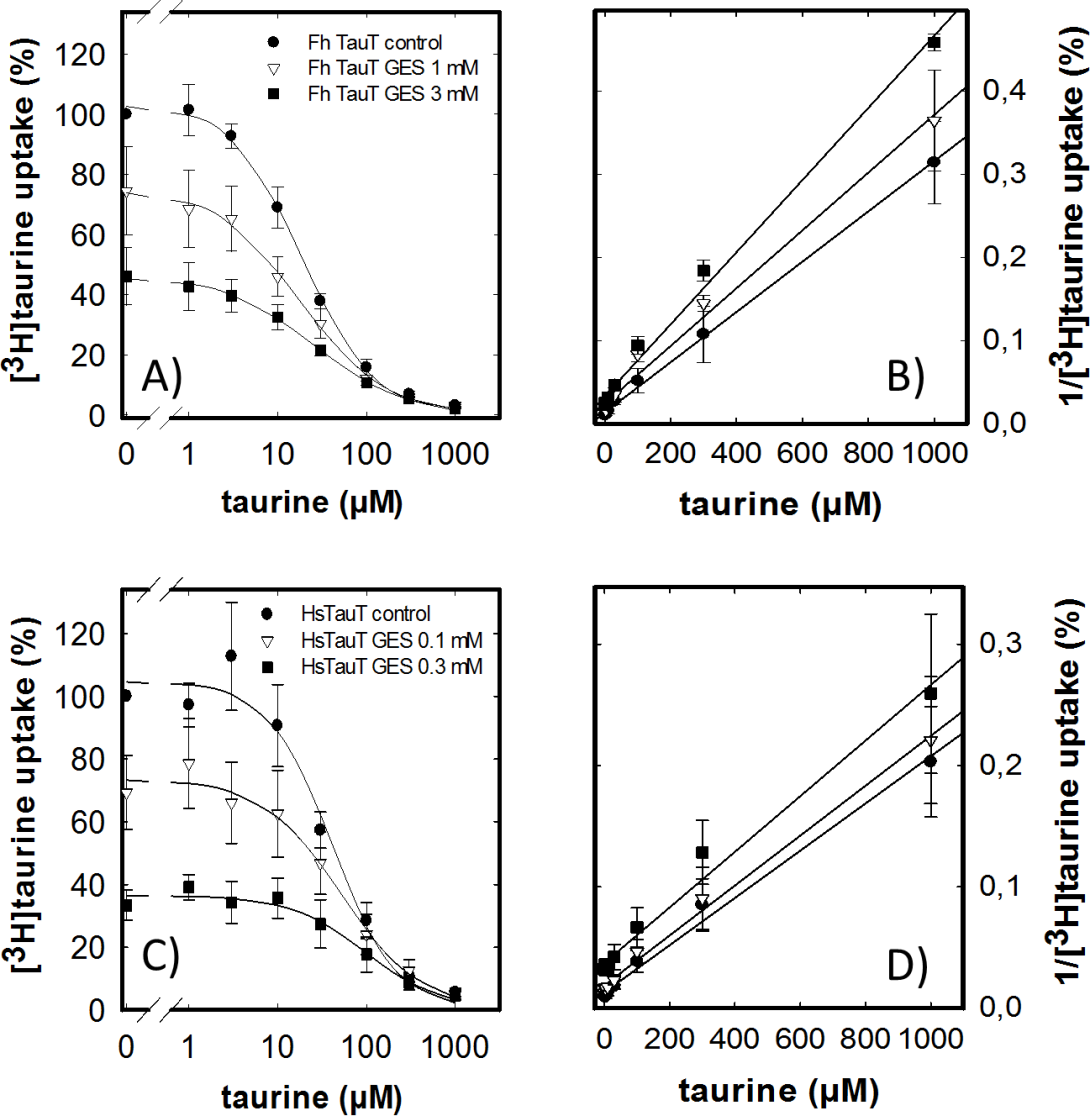
Inhibition of [^3^H]taurine uptake by unlabeled taurine in the absence and presence of GES. HEK293 cells (1*10^5^ cells/well) stably expressing the *F*. *hepatica* (A, B) or the human taurine transporter (C, D) were incubated for 10 min in the presence of 0.1 μM [^3^H]taurine and of the indicated concentrations of unlabeled taurine and of GES. Data represent means ± S.D. from three independent experiments carried out in duplicate. The solid lines in panels A and C were drawn by fitting the data to a monophasic inhibition curve. In panels, B and D, the data shown in panels A and C, respectively, were replotted in Dixon plots to visualize the non-competitive and the competitive inhibitory action of GES by the intersecting lines (B) and the parallel lines (D), respectively.

The number of compounds which act as specific inhibitors of taurine uptake is very limited. Because of the close relation between GABA- and taurine transporters, we examined several compounds, which act as inhibitors of GABA uptake or of GABA degradation (e.g., nipecotic acid, tiagabine, vigabatrin), but these failed to inhibit the *F*. *hepatica* transporter. In fact, the only compound other than GABA and β-alanine (cf. Fig 4) was guanidinoethyl sulfonate (taurocyamine or GES). This compound was classified as a competitive inhibitor of mammalian taurine transporters [43]. We verified the mode of inhibition by determining uptake of [^3^H]taurine by cells expressing the *F*. *hepatica* (Fig 6A) and the human transporter (Fig 6C) in the presence of increasing concentrations of unlabeled taurine and of GES: in cells expressing the human transporter, the action of GES was consistent with competitive inhibition, because the IC_50_ of unlabeled taurine was progressively shifted to the right (Fig 6C; IC_50_ = 41.2 ± 8.2 μM, 52.1 ± 9.5 μM and 98.9 ± 20.1 μM in the absence and presence of 0.1 and 0.3 mM GES, respectively). Accordingly, when the data were replotted in a Dixon plot, the resulting lines were parallel indicating that binding of taurine and GES was mutually exclusive (Fig 6D). The affinity of the *F*. *hepatica* transporter for GES was about ten-fold lower than that of the human transporter; this can be seen by comparing the uptake in the absence of unlabeled taurine in Fig 6A and 6C, which was about 75% and 40% in the presence of 1 mM and 3 mM GES, respectively, for the *F*. *hepatica* transporter (Fig 6A). Equivalent residual transport was seen at 0.1 and 0.3 mM GES with the human transporter (Fig 6C). In addition, GES was a non-competitive inhibitor of taurine uptake by the *F*. *hepatica* transporter: the IC_50_ of unlabeled taurine were not shifted to the right in the presence of GES (Fig 6A; IC_50_ = 11.7 ±4.1 μM, 18.7 ± 6.7 μM, and 16.0 ± 4.2 μM in the absence and presence of 1 and 3 mM GES, respectively). Transformation of these data into a Dixon plot produced a family of intersecting lines. This indicates a non-competitive inhibition, where taurine and GES can be bound simultaneously.

### Detection of the *F*. *hepatica* taurine transporter by immunoblotting

**Fig 7:**
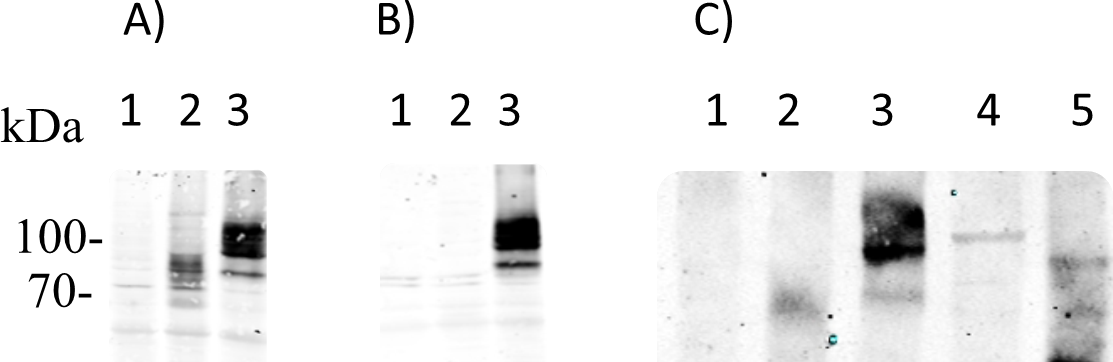
**Detection by immunoblotting of the *F*. *hepatica* taurine transporter after heterologous expression (A, B) and in lysates prepared from adult flukes (C). A&B:** lysates (20 μg/lane) were prepared from untransfected HEK293 cells (lane 1), from HEK293 cells stably expressing the *F*. *hepatica* taurine transporter (lane 2) and a YFP-tagged version of the transporter (lane 3). After electrophoretic separation on an SDS-polyacrylamide gel, the proteins were transferred to a nitrocellulose membrane, which was probed with an affinity purified rabbit polyclonal antibody raised against the N-terminus (A) and an anti-GFP antibody (B). **C:** lysates (1 μg) prepared from untransfected HEK293 cells (lane 1) or from HEK293 expressing the untagged (lane 2) and the YFP-tagged *F*. *hepatica* taurine transporter (lane 3) were resolved by denaturing gel electrophoresis together with detergent extracts (40 μg) of adult fluke homogenates (lane 4) and of tegumental particulate material (lane 5). After transfer to nitrocellulose membranes, the immunoreactive bands were visualized as described for panels A & B. The immunoblots are representative of four independent experiments.

We raised a rabbit polyclonal antiserum against the N-terminus of the *F*. *hepatica* taurine transporter using a purified fusion protein comprising maltose-binding protein (MBP) and the N-terminus (i.e., residues R^2^-R^56^) as the immunogen. We also generated the fusion of glutathione-S-transferase (GST) and the N-terminus of the *F*. *hepatica* taurine transporter. The purified GST-fusion protein was bound to GSH-sepharose 4B, which allowed for the enrichment of the taurine transporter-specific antibodies by affinity purification. The resulting antibody preparation was tested on cell lysates prepared from untransfected HEK293 cells and from cells expressing either a YFP-tagged version of the *F*. *hepatica* taurine transporter or the non-tagged protein (Fig 7A). Consistent with the multiple glycosylation sites of the *F*. *hepatica* taurine transporter (*cf*. Fig 1), the antibody detected several bands of ~70 kDa and ~95 kDa in cell lysates containing the un-tagged (Fig 7A, lane 2) and the YFP-tagged version (Fig 7A, lane 3), respectively. These immunoreactive bands were not seen in lysates prepared from untransfected cells (Fig 7A, lane 1). As an additional control, we also used an antibody against GFP for detection of the YFP-tagged transporter (Fig 7B): the immunoreactive bands, which were visualized by the anti-GFP antibody, were identical to those detected by the antibody raised against the N-terminus (cf. right-hand lanes in Fig 7A and 7B).

Homogenates were prepared from adult *F*. *hepatica*; in addition, we enriched for tegumental cell membranes by subjecting adult flukes to a freeze-thaw cycle combined with vigorous vortexing. This procedure allows for collecting the particulate material from the tegument from the adult flukes and for the substantial enrichment of surface markers [43–45]. Although the sensitivity of the immunoblot sufficed to detect the heterologously expressed transporter in 1 μg of cell lysate (Fig 7C, lanes 2 and 3 for the untagged and YFP-tagged transporter respectively), it was not possible to detect any immunoreactive material of the expected molecular mass in lysates from adult flukes (Fig 7C, lane 4). However, the preparation enriched in tegumental membranes contained immunoreactivity for the taurine transporter (Fig 7C, lane 5).

### Survival of *F*. *hepatica*

**Fig 8:**
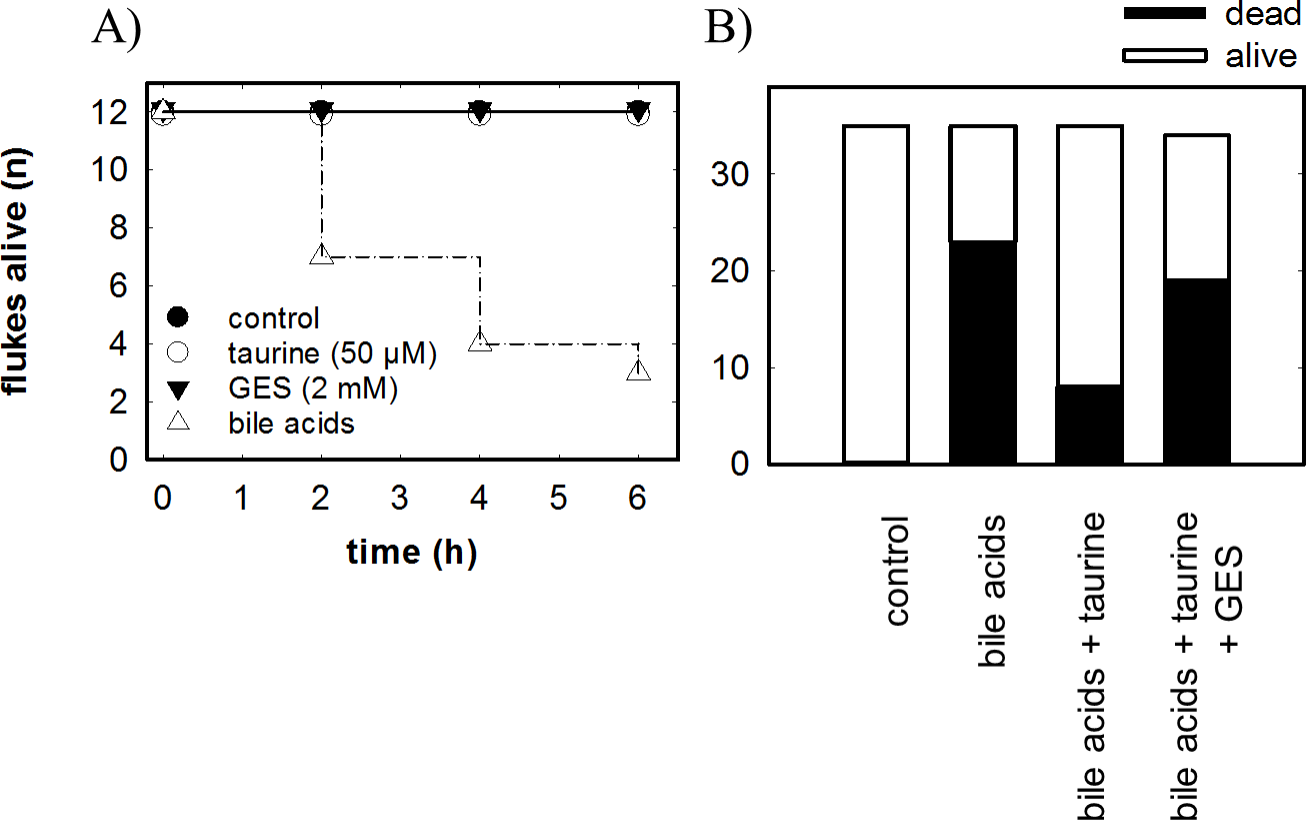
Survival of adult *F*. *hepatica* in bile acid-containing medium in the presence and absence of taurine. **A:** Adult flukes (12 flukes/condition in individual wells) were incubated at 37°C and 5% CO_2_ in a humidified atmosphere in Hédon Feig medium in the absence (control, closed circles) or presence of 50 μM taurine (closed circle), of 2 mM GES (GES, closed triangle) or of bile acids (closed triangle, 5 mM cholic acid, 1.5 mM deoxycholic acid and 0.5 mM chenodeoxycholic acid). The number of dead flukes was recorded at the indicated time points. **B:** Adult flukes were incubated at 37°C and 5% CO_2_ in a humidified atmosphere in Hédon Feig medium in the absence (control) or presence of bile acids (5 mM cholic acid, 1.5 mM deoxycholic acid and 0.5 mM chenodeoxycholic acid). Where indicated, the medium contained, in addition, 50 μM taurine or the combination of 50 μM taurine and 2 mM GES (GES) for 4h. The number of dead flukes was recorded at the end of the incubation period. The data are from three independent experiments with 11 to 12 flukes/condition. Survival in the presence of bile acids and taurine differed significantly from that observed in the sole presence of bile acids (p = 0.0006, Fisher's exact test) and in the presence of the combination of bile acids, taurine and GES (p = 0.0068, Fisher's exact test), but there was no significant difference between survival in the presence of bile acids and of the combination of bile acids, taurine and GES (p = 0.464). Thus, after Bonferroni correction for multiple testing (with α≤0.016), taurine afforded a statistically significant protection against bile acids.

Living adult *F*. *hepatica* can survive more than 14 days in Hédon-Fleig medium [46]. It is possible to maintain flukes for even longer periods—i.e. up to 6 months—in the presence of chicken serum [47]. However, chicken serum contains undefined amounts of taurine, albumin and lipoproteins. Thus, we carried out our experiments in Hédon-Fleig medium without any addition of chicken serum to preclude possible interference by carry-over of taurine, drug binding to albumin and binding of bile acids to lipoproteins. Accordingly, we placed adult flukes into the Hédon-Fleig medium. Under control conditions and in the presence of 50 μM taurine or of 2 mM GES, all flukes were alive and motile for at least 6 h (Fig 8A). In contrast, if flukes were exposed to a mixture of unconjugated bile acids, i.e. cholic acid, chenodeoxycholic acid and deoxycholic acid as found in bovine bile [48], flukes were progressively killed such that their median survival was about 3 hours (open triangles in Fig 8A). We exposed a total number of 35 flukes to bile acids for 4 h, which was lethal for about two thirds (second bar in Fig 8B). Flukes were protected against the deleterious effect of bile acids by the presence of 50 μM taurine in the medium, which afforded a statistically significant protection: survival increased to about 80% in the presence of bile acids and taurine (third bar in Fig 8B). This protective effect was abolished, if GES was also present in the medium, with only 44% surviving (fourth bar in Fig 8B). Consistent with the hypothesis that the taurine transporter is important for survival of the flukes in bile, we found that the transcripts for the taurine transporters are low in the early life stages (fertilized egg, metacercariae) and increase substantially during maturation of juvenile flukes (S5 Fig).

## Discussion

The SLC6 transporter family has four branches: (i) the neurotransmitter transporters (i.e., transporters for dopamine, norepinephrine and serotonin), (ii) the glycine and proline transporters, (iii) the amino acid transporters SLC6A15 to SLC6A20 and (iv) the GABA-transporter subfamily, which mediates the uptake of GABA, betaine, creatine and taurine [49]. The evolutionary history of this latter branch was traced by sequence comparisons and by examining chromosomal synteny [50]; the following sequence of events was proposed: gene duplication of the ancestral transporter gene gave rise to the GABA-transporter-1 (GAT-1/SLC6A1) and the creatine transporter-1 (CT1/SLC6A8). The taurine transporter (TauT/SLC6A6) is the third gene, which appeared during evolution, presumably by duplication of the creatine transporter-1. In chordates, GAT3 (SLC6A11) arose from TauT. GAT2/BGT1 (betaine/GABA-transporter-1, SLC6A12) and GAT4 (SLC6A12) were later additions during vertebrate evolution. Here we identified an SLC6 family member in *F*. *hepatica*. We classified this transporter as a taurine transporter based on both its sequence homology with the other protostomial taurine transporter, for which the biochemical activity has been verified, i.e. the TauT of *B*. *septemdierum* [33] and an in-depth characterization of its biochemical specificity: our data unequivocally show that taurine is the preferred substrate of the transporter. In fact, the affinities of the alternative substrates β-alanine and GABA were about 50- and 350-fold lower, respectively, than that of taurine. Taurine uptake was dependent on both, Na^+^ and Cl^-^. This was to be expected: with the notable exception of SLC6A17, which is thought to function as a Na^+^-dependent vesicular amino acid transporter [51,52], all eukaryotic SLC6 transporters function as Na^+^/Cl^—^dependent plasma membrane transporters. The majority relies on a stoichiometry of two sodium ions and one chloride ion. This was also true for the *F*. *hepatica* taurine transporter: chloride enhanced transport velocity in a hyperbolic fashion, but sodium stimulated taurine uptake with a sigmoidal concentration-response curve resulting in a Hill-coefficient close to 2. This 2:1 stoichiometry is also consistent with the finding that the residues, which define the binding sites for the two sodium ions and the chloride ion are conserved in the *F*. *hepatica* taurine transporter.

Taken together, our observations showed that the *F*. *hepatica* taurine transporter differed from its human orthologue in several respects: (i) the chloride affinity of the *F*. *hepatica* taurine transporter was substantially higher than that of the human transporter. From a teleological perspective, this finding can be rationalized as an adaptation to the ionic composition of the bile: the chloride concentration in mammalian bile is substantially lower than that of plasma [53]. Thus, an affinity in the range of 12 mM assures that the fluke transporter operates at close to saturation of the chloride site regardless of the changes in ionic composition resulting from hormonal stimulation of bile flow [53]. (ii) Similarly, the K_M_ of the *F*. *hepatica* transporter for taurine was lower than that of the human orthologue. The concentration of taurine in human plasma is in the range of 50 μM [54]; thus the K_M_ of the human taurine transporter is close to the extracellular levels. Although taurine was originally identified in ox bile [55], the concentration of free taurine in bile is—to the best of our knowledge—not known. We suspect that the higher affinity of the *F*. *hepatica* taurine transporter reflects an adaptation to the lower concentration of taurine in bile. (iii) The pharmacology of taurine transporters has not yet been explored in depth. In spite of the limited availability of inhibitors, our observations show that the *F*. *hepatica* transporter differs substantially from that of the human taurine transporter: GABA and β-alanine were about 3 and 5-fold less potent, respectively, in inhibiting the *F*. *hepatica* than the human transporter. The affinity of GES for the *F*. *hepatica* was also lower. Importantly, GES was a non-competitive inhibitor of the *F*. *hepatica* transporter. The non-competitive mode of inhibition can be rationalized by taking into account that SLC6 transporters harbor two binding sites, namely the vestibular S2 site and substrate binding site proper, which is referred to as S1 site [49,56]. The non-competitive action may arise if GES binds preferentially to the S2 site: occupancy of the vestibular site and of the substrate binding site proper is not mutually exclusive and results in non-competitive inhibition [57]. At the very least, our observations justify the assumption that the taurine transporter of *F*. *hepatica* differs enough to allow for the development of selective specific inhibitors, which target the fluke transporter but not the mammalian orthologue.

The mechanism by which taurine accumulation protects *F*. *hepatica* from bile acid toxicity is not known. In mammals, taurine deficiency or genetic deletion of the taurine transporter results in pleiotropic effects, which culminate in retinal degeneration, liver and kidney disease, skeletal muscle wasting, etc. [58,59]. It is generally accepted that taurine is an osmolyte, which protects cells against various types of stress at least in part by stabilizing proteins against denaturation [60]. Bile acids, in particular deoxycholic acid, are chaotropic and promote unfolding of proteins [61]. Thus, it is plausible to posit that taurine accumulation in flukes is a safeguarding mechanism, which blunts bile acid toxicity. The alternative hypothesis is to assume that flukes use taurine as a substrate to conjugate free bile acids; the resulting conjugated bile acids are subsequently re-exported into the bile by an ABC-transporter. This hypothetical mechanism of detoxification requires at least two components, (i) a bile acid-CoA: amino acid N-acyltransferase and (ii) a bile salt export pump. In fact, adult *F*. *hepatica* express many ABC transporters. Previously, a murine monoclonal antibody, which had been raised against a peptide derived from the first nucleotide binding domain (Y^705^-K^718^) of human ABC-B11, was used to immunoblot lysates of adult *F*. *hepatica*: an immunoreactive band was detected, albeit of only 80 kDa, which is half the size of the human orthologue [62]. Hence, it is not clear, if adult flukes express a bile salt export pump. In addition, we failed to find any evidence for the presence of a bile acid-CoA: amino acid N-acyltransferase by analyzing the deposited genomic sequence [23,24]. Regardless of the underlying mechanism, our observations suggest that the taurine transporter is essential for the survival of *F*. *hepatica* in hostile environments. The protective action of intracellular taurine is presumably the driving force for the early appearance of the taurine transporter during evolution [50]. We suspect that the hypothetical GABA-transporter-2 of *C*. *sinensis* is, in fact, also a taurine transporter. The same is likely to be true for the other trematode SLC6 transporters in Fig 2, i.e., *O*. *viverrini* hypothetical protein, the *S*. *haematobium* GABA-transporter-2 and the *S*. *mansoni* serotonin transporter. Hence, designing specific inhibitors may also be of interest to explore the role of these transporters in the biology of these parasites. Parasites are by definition auxotrophic. Thus they must rely on transporters to obtain nutrients and other solutes, which they require for survival. Thus solute carriers are likely to represent drug targets, which allow for the control of parasitic disease. Our observations show that the taurine transporter is essential for survival of *F*. *hepatica* in the presence of bile acids. We anticipate that inhibitors of the taurine transporter may not only be useful to eliminate the adult stage of *F*. *hepatica* from the liver of affected individuals but also of the other liver flukes, which infest people, i.e. *C*. *sinensis* and *Opisthorchis viverinni*.

## Materials and methods

### Ethics statement

Rabbits were immunized at the “Department für Biomedizinische Forschung, Medical University of Vienna”. This institution holds a permission (BMWF-66.009/0266-II/3b/2013) by the Austrian Ministry of Science to immunize rabbits according to §26 Austrian Animal Testing Law of 2012 (TVG 2012 [63]). All efforts were made to minimize animal suffering and to reduce the number of animals used.

### Reagents and chemicals

If not stated otherwise, cell culture plastic dishes and pipettes were from Sarstedt AG&Co., Nuembrecht, Germany, chemical and reagents including cell culture media were from Sigma Aldrich.

### Collection and processing of adult flukes

According to European Regulation (EC 854/2004) [64], the livers of slaughtered cattle have to be inspected by veterinarians for a possible infestation of animals with parasites using visual, palpation and incision inspection [65]. Based on this surveillance, livers associated with liver flukes are discarded together with other abnormalities [65]. Before this, we inspected these livers for the presence of *F*. *hepatica*. Adult flukes were collected from the infected bile ducts of freshly slaughtered cattle in local abattoirs (Eschenau & Salzburg, Austria) and washed with phosphate-buffered saline (PBS; composition: 2.7 mM KCl, 1.5 mM KH_2_PO_4_, 137 mM NaCl, 4.3 mM Na_2_HPO_4_ x 2H_2_O, pH 7.3) and either maintained in Hedon Fleig solution (120.7 mM NaCl, 4 mM KCl, 1.9 mM MgSO_4_, 0.9 mM CaCl_2_, 18.5 mM NaHCO_3_, 10 mM HEPES, 15 mM D-glucose adjusted pH to 7.3) [46] or frozen in liquid nitrogen and stored at -80°C.

### PCR amplification of cDNA and cloning

Total RNA was isolated from freshly isolated adult flukes using Trizol (Sigma Aldrich, Mannheim, Germany) Reverse transcription was performed using “Transcriptor High Fidelity cDNA Synthesis Kit (Roche Diagnostics GmbH, Mannheim, Germany), using either gene specific primers or oligo dT primers. Starting from a previously deposited cDNA sequence 5’ and 3’ ends of the cDNA were identified via RACE (rapid amplification of cDNA ends) technology, using 5’/3’ RACE Kit, 2nd Generation (Roche Diagnostics GmbH, Mannheim). Recognition sites for XhoI and KpnI were added to the full-length cDNA of FhTauT by PCR. The resulting PCR product was cloned via XhoI and KpnI to peYFP-C1 (Takara Bio Europe, France) generating a transporter tagged with a yellow fluorescent protein at its N-terminus. To generate a non-tagged transporter, FhTauT was also cloned to pcDNA3.1 (Invitrogen, Carlsbad, USA). HsTauT was cloned in a similar way to peCFP-C1 via BamHI and HindIII thereby generating a transporter tagged with the cyan fluorescent protein. Generated sequences were validated by Sanger sequencing (LGC Genomics, Berlin, Germany). The cDNA sequence of FhTauT was deposited at GenBank (NCBI, Bethesda, USA) under the accession number: MG674191.

### Bioinformatic analysis

The partial initial sequence of Fh TauT mRNA was obtained from the database published by Gasser et al. [22] and full-length mRNA was obtained by applying RACE techniques (for see PCR amplification of cDNA and cloning). The genomic sequence was obtained from the database WormBase (https://parasite.wormbase.org) [23] and is based on the published *F*. *hepatica* genome (PRJEB6687) [66].

We analyzed the expression of FhTauT in various life stages by searching the compilation of RNAseq data deposited in the WormBase with the built-in BLAST tool (PRJEB6904) [66]. We focused exclusively on the gene encoding FhTauT and extracted the expression levels of various exons at individual lifecycle stages of the parasite. We normalized the number of reads per million base pairs to that seen in fertilized *Fasciola* eggs (expression levels at this stage = 1, hence log2 = 0).

Exon-intron boundaries of the final extended FhTauT sequence were detected using the splign web interface (https://www.ncbi.nlm.nih.gov/sutils/splign/splign.cgi) [67]. The visualization of exons and introns organization in the genome was done by Exon-Intron Graphic Maker (http://wormweb.org/exonintron).

Orthologous sequences were identified using the basic local alignment tool (BLAST) web interface provided by NCBI using non-redundant protein sequences database (nr) [68]. Sequence alignment and phylogenetic analysis were performed using MEGA 7 [34] Multiple Sequence Comparison by Log-Expectation (MUSCLE [69]) and Clustal W [70] respectively. The phylogenetic trees were using the neighbor-joining method [71]. The results were displayed using ENDscript (http://endscript.ibcp.fr/) [72]. Accession numbers for sequences included can be found in the Material & Methods section.

For the construction of a phylogenetic tree (S3 Fig) covering parasites only, orthologous sequences of Platyhelminthes deposited in the WormBase (except *Protopolystoma xenopodis*, *Trichobilharzia regenti* and *Schmidtea mediterranea* which has only truncated versions available) were exported into Molecular Evolutionary Genetics Analysis (MEGA) software version 7 based on their best E value, score and identity (S6 Tab.), and aligned with ClustalW. In addition a sequence from *F*. *gigantica* was included, obtained from the database published by Gasser et al. [22]. Phylogenetic analysis was performed using MEGA 7 [34]. (neighbor-joining, 1000-replicate, bootstrap). The amino acid data were corrected for a gamma distribution (level set at 1.0) and with a Poisson correction.

The NetNGlyc 1.0 Server (www.cbs.dtu.dk) was used to search for putative N-glycosylation sites in the final sequence of FhTauT [73]. We used the crystal structure of Hs SERT, derived from Coleman et al. (PDB ID:5I6X, DOI: 10.2210/pdb5i6x/pdb) to compare the protein structure with FhTauT and other transporters [25].

### Cell line generation and uptake measurements

Human embryonic kidney-293 (HEK293 (ATCC CRL-1573, LGC standards Wesel, Germany)) cells, were grown in Dulbecco`s modified Eagle medium (DMEM) supplemented with 10% fetal bovine serum (FCS Nuaille, France), 100 units/ml penicillin and 100 μg/ml streptomycin at 37°C and with 5% CO_2_ in a humidified incubator. Cells were transfected with plasmids encoding fluorescently tagged or untagged versions of *F*. *hepatica* (FhTauT) and H. sapiens (HsTauT) taurine transporter using Jet Prime transfection reagent (Polyplus-transfection, France). To generate monoclonal cell lines stably expressing the transporters, transfected cells were subjected to and selected with the concentration of 0.2 mg/ml geneticin (G418) for 10 days. Surviving cells forming colonies were separated and analyzed for membrane localization of transporters and transport of taurine. After the selection process cells were kept at 50 μg/ml G418 to keep the selection pressure.

For saturation uptake experiments, cells were plated the day before the experiment at a density of about 50,000 cells/well onto poly (D-lysine)-coated 48-well culture plates (CytoOne, USA). On the day of the experiment, cells were washed with prewarmed Krebs-HEPES buffer twice (KHB) (120 mM NaCl, 3 mM KCl, 2 mM CaCl_2_, 2 mM MgCl_2_, 20 mM glucose, 10 mM HEPES, pH 7.3). Then, cells were incubated in KHB containing tracer amounts (0.1 μM) of [^3^H]taurine, (19.1 Ci/mmol) (Perkin Elmer, USA) together with increasing concentrations of non-labeled taurine 10 min. The reaction was terminated after 10 minutes by removing the medium followed by rapid rinsing of cells with ice-cold assay buffer. Subsequently, cells were lysed with 0.5 ml of 1% sodium dodecyl sulfate (SDS) and transferred into scintillation vials for liquid scintillation counting. Non-specific uptake was determined by incubating cells in the presence of the blocker β-alanine (100 mM) before and during the experiment and subtracted from uptake values. Data were fit to a Michaelis-Menten equation.

Chloride-free KHB was prepared to examine the chloride dependence of both, FhTauT and HsTauT using acetate salts instead of chloride salts. Likewise, NaCl was replaced by choline chloride to examine the sodium requirement. Uptake experiments were performed as described above using 0.1 μM [^3^H]taurine in sodium- and chloride-free modified KHB, respectively, mixed with increasing amounts of NaCl containing KHB thereby varying Na^+^ and respectively Cl^-^ concentration from 0–120 mM. The inhibition experiments were performed in analogous manner with 0.1 μM [^3^H]taurine and the logarithmically spaced concentrations of inhibitors.

GABA uptake was assessed by incubating HEK293 cells (10^5^/ well) stably expressing the human GABA-transporter-1 (HsGAT1, diamonds), the *F*. *hepatica* taurine transporter (FhTauT, circles) and the human taurine transporter (HsTauT, triangles) in KHB containing concentrations of [^3^H]GABA covering the range of 0.3 μM to 3 mM for three minutes. The amount of labeled [^3^H]GABA was kept constant and the specific activity was progressively diluted by the addition of unlabeled GABA (from 9 Ci/mmol to 9 Ci/mol). The reaction was stopped by the addition of ice-cold KHB followed by three rapid washes. Cells were detached and the accumulated radioactivity was determined by liquid scintillation counting [74].

### Confocal microscopy

For confocal imaging, HEK293 cells stably expressing YFP-FhTauT and CFP-HsTauT were seeded onto poly-D-lysine–coated glass-bottomed chambers 24 h prior to the experiment. Cells were imaged using a 60x oil immersion objective (Plan Apo VC, Nikon, Austria) on a confocal laser scanning microscope (A1R+, Nikon, Vienna, Austria). The fluorophores YFP and CFP were excited using a 488 nm and 403.5 nm laser line, respectively, at 1–5% of maximal intensity. Emission of CFP and of YFP was detected with a standard PMT (photomultiplier tube) detector equipped with a 435 nm emission filter (50 nm bandpass) and a GaAsP detector equipped with a 525 nm filter (50 nm bandpass). The cell membrane was visualized by incubating the cells in a trypan blue solution (0.05%) for 5 min. Fluorescence of trypan blue was excited using a 561.9 nm laser line, the emission was detected with a GaAsP detector with a 595 nm filter (50 nm bandpass).

### Sample preparation and western blotting

Freshly isolated flukes were washed twice with PBS. Afterwards, flukes were homogenized at 4°C in HME buffer (10 mM HEPES, 1 mM MgCl_2_, 0.1 mM EDTA, and pH 7.4) using an Ultra-Turrax dispersing instrument (Janke&Kunkel, IKA-WERK, Germany). The sheared flukes underwent two freeze/thaw cycles in liquid nitrogen. After repeated sonication, the lysate was centrifuged at 10,000 g for 15 minutes at 4°C. The pellet was resuspended in 5 ml of buffer (1 mM EDTA, 0.1% sodium deoxycholate, 0.1% SDS, 140 mM NaCl, 10 mM Tris.Cl (pH 7.4.) supplemented with one tablet of protease inhibitors per 20 ml (cOmplete Protease Inhibitor Cocktail, Roche)), incubated overnight on a rocking platform and centrifuged at 13,000 g at 4°C for 10 min. Supernatants from cell lysates were mixed with Laemmli buffer (0.1% 2-mercaptoethanol, 0.01% bromophenol blue, 10% glycerol, 2% SDS, 62.5 mM Tris.HCl, pH 6.8) and used for SDS polyacrylamide gel electrophoresis.

For *Fasciola* tegument preparation we used the “freeze-thaw and vortex” method described by Roberts et al. [44] with minor modifications. About 5 g adult flukes (corresponding to 20 flukes) were flash frozen in liquid nitrogen and then thawed on ice in 5 ml of cold RPMI-1640 including protease inhibitors as described above. The tegument was detached by vigorous vortexing for 1 min from the bodies of the flukes, and the supernatant was filtered through a metal sieve. The denuded bodies were pelleted by centrifugation at 1000g for 30 min at 4°C. The resulted pellet was resuspended in HME buffer, and freeze-thaw was done twice. The produced sample was sonicated thrice. The sample was centrifuged at 40,000g for 2 hours at 4°C. The pellet was solubilized with Laemmli buffer.

Proteins were separated via SDS-PAGE electrophoresis and transferred from the gel to a nitrocellulose membrane. Membranes were blocked in Tris-buffered saline (TBS) containing 0.1–0.5% Tween 20 and 3% bovine serum albumin (BSA) or 3% skimmed milk. For detection, the purified antibody against N-terminus was used at a dilution from 1:100 to 1:50. The following commercial antibodies were used-rabbit polyclonal anti-GFP (Ab290 Abcam; 1/5000), anti-rabbit IRDye 680RD or anti-rabbit IRDye 800CW (LiCOR Biosciences Fluorescence signal on membranes was detected by Licor Odyssey CLx, (Imaging System).

### Generation of polyclonal antibodies

Two rabbits were immunized with a fusion protein construct consisting of maltose binding protein, and the amino-terminus of FhTauT (RQEFSFPSKSRTELAATSSIHPV FEVKDECSIIPVSSSLANKKEVEKSPREQWKR). The immunization was carried out at the Medical Unversity of Vienna, Div. Laboratory Animal Science and Genetics, Himberg, Austria. Short: The antigen solutions for the immunization of one rabbit were prepared by emulsifying 100 μg MBP fusion protein in 750 μl 1x PBS (aqueous solution) and 1 ml (in)complete Freund’s adjuvant (oleaginous solution). The emulsions were kept at 4°C, and the immunization was performed within 1 h. Antibodies were affinity purified out of serum from immunized rabbits. For this 6-His and glutathione-S-transferase (GST) was fused to the same N-terminal peptide of FhTauT as mentioned above and expressed in *E*. *coli* BL21. Subsequently, the protein eluted by cOmplete His-Tag Purification Resin (Sigma Aldrich, Mannheim, Germany) under denaturing conditions and urea content was reduced from 8 M to 5 M with a saline solution. To optimize the coupling to Affi-gel 10 (BioRad, California, USA), eluates were pH adjusted based on their isoelectric point titrating either NaOH or HCl. To block free binding sites of the Affi-gel 10, 100 μl 1 M ethanolamine pH 8.0 per ml Affi-gel 10 suspension were added for 1 h at room temperature. Antibodies were eluted with alkaline elution buffer (pH 11.5). 1 ml fractions were collected in Eppendorf tubes prefilled with 100 μl acidic neutralization buffer (pH 2.45). Protein-containing fractions were pooled, and the protein concentration was determined using the bicinchoninic acid (BCA) protein assay reagent (Pierce, ThermoFisher Scientific).

### Survival assay

*F*. *hepatica* was isolated from the bile ducts of cows slaughtered at Alpenrind GmbH (Salzburg, Austria). Flukes were kept in modified Hedon Fleig`s solution at 37°C and 5% CO_2_ in a humidified atmosphere. Before the experiment, flukes were maintained at 37°C for three days after extraction to clear them from bile acids. Living flukes (judged by observing movement after gently tapping them with metal forceps) were transferred into flasks containing either Hedon Fleig`s Solution alone or Hedon Fleig`s Solution supplemented with combinations of bile acids (5 mM cholic acid (CA), 1.5 mM deoxycholic acid (DCA) and 0.2 mM chenodeoxycholic acid (CDC)), 50 μM taurine and 2 mM guanidinoethyl sulfonate (GES). Survival of flukes was determined again after four hours of treatment.

### Statistics

Data are expressed as arithmetic means ± S.E. Statistics Nonlinear regression analysis was carried out for uptake assays to determine the Km values using the Sigma Plot software program (version 12.5; Systat Software, Chicago, IL, USA) or GraphPad Prism software (version 5.04, GraphPad Software, La Jolla California, USA). Hill coefficients for Na^+^ and Cl^-^ dependent experiments were obtained from the sigmoidal curves using the softwares as noted above.

### Accession numbers

To generate a phylogenetic tree (Fig 2) we used the following proteins: *C*. *sinensis*, accession no: GAA52609.1; *O*. *viverrini*, XP_009175490.1; *S*. *mansoni*, XP_018646439.1; *S*. *haematobium*, XP_012792810.1; *B*. *septemdierum*, BAF95543.1; *D*. *melonagaster*, NP_651930.2; *H*. *sapiens*, P31645.1; *H*. *sapiens*, NP_003034.2; *H*. *sapiens* NP_057699.2.

## Supporting information

S1 Fig **Primary structure of the *F*. *hepatica* taurine transporter (FhTauT) (A) and exon-intron structure of its gene (B).** A: The cDNA of FhTauT and its conceptual translation. Amino acids are shown in three letter code. The blue lines mark the borders of consecutive exons, which are numbered (bold numbers). B: Exons and introns are indicated by boxes and lines, respectively. Coding sequences are shown as full boxes; the 5'- and 3'-untranslated regions are represented as white boxes. The analysis was performed by comparing the cDNA with the genomic sequences deposited in the worm base (http://parasite.wormbase.org/) using the Splign web tool (https://www.ncbi.nlm.nih.gov/sutils/splign/splign.cgi). The figure was generated by Exon-Intron Graphic Maker (http://www.wormweb.org/exonintron). The scale bar represents 10 kb.
(TIF)

S2 Fig Comparison of the amino acid sequence of the taurine transporter of *F*. *hepatica* and *F*. *gigantica*. The sequence of the *F*. *gigantica* transporter was retrieved from the transcriptome deposited in (http://bioinfosecond.vet.unimelb.edu.au/index.html) [ref. 22] by BLAST search and aligned with that of the *F*. *hepatica* TauT by using Clustal Omega (version 1.2.4). The two proteins differ in 12 out of 647 positions (98% identity). Identical residues are marked by asterisks, highly (3) and less well-conserved residues (2) by colons and dots, respectively, and dissimilar residues (7) by blank spaces. The differences cluster in the N- and C-termini (i.e., before residue 57 and after residue 571); the other four changes are in intracellular loop 5 (IL5) and extracellular loop 6 (EL6).
(TIF)

S3 Fig Phylogenetic comparison of the amino acid sequence of the taurine transporter of *F*. *hepatica* with transporters of other Platyhelminthes. The phylogenetic tree was constructed with the neighbor-joining method implemented in MEGA 7 [34]: the amino acid sequence of FhTauT was compared to that of orthologues found in Platyhelminthes available in WormBase (for accession numbers of sequences see S1 Table.). We also found an orthologue in *Protopolystoma xenopodis*, *Trichobilharzia regenti* and in *Schmidtea mediterranea*, but we did not include these, because only very short sequences were available. Bootstrap valued (1000 replicates, Poisson correction) are shown next to the branches. The scale bar reflects the likelihood that a change in amino acid occurred on any given branch. Digenean transporters/contigs are highlighted in the gray area, where the underlined sequence is the one originally derived from NCBI, and used to generate the tree in Fig 2. The yellow area marks the transporters/contigs from other Platyhelminthes.
(TIF)

S4 Fig [^3^H]GABA uptake by the human GABA-transporter-1, the *F*. *hepatica taurine transporter* and the human taurine transporter. HEK293 cells (10^5^/ well) stably expressing the human GABA-transporter-1 (HsGAT1, diamonds), the *F*. *hepatica* taurine transporter (FhTauT, circles) and the human taurine transporter (HsTauT, triangles) were incubated in the presence of the indicated concentrations of [^3^H]GABA. The specific activity was progressively diluted by addition of unlabeled GABA (from 9 Ci/mmol to 9 Ci/mol). After three minutes the reaction was stopped and the accumulated radioactivity was determined by liquid scintillation counting. Data are means ± S.D. of at least two independent experiments performed in triplicate.
(TIF)

S5 Fig Developmental expression of FhTauT. RNAseq data were extracted from the WormBase with the built-in BLAST tool (PRJEB6904). The expression level of FhTauT was quantified by the number of reads per million base pairs in different developmental stages (i.e. eggs, metacercariae, newly emerged juveniles after 1,3 and 24hrs, juveniles and adults) [66]. The reads were normalized to the expression levels in eggs (= 1) and plotted as fold-increase over this level (log2).
(TIF)

S1 Table Sequences of transporters from Platyhelminthes used for phylogenetic analysis. **“**Genome” represents the source of organisms, the column “subject name/accession no” provides the accession numbers from the respective source, “subject description” gives information about the possible function of the protein as indicated in the data base; “source” gives the digital address from where the sequence was retrieved.
(XLSX)
