# Supplementary figures and images for: Identification and characterization of the Fasciola hepatica sodium- and chloride-dependent taurine transporter
Source: PLoS Negl Trop Dis. 2018 Apr 27;12(4):e0006428. doi: 10.1371/journal.pntd.0006428 (PMC5942844; doi:10.1371/journal.pntd.0006428)

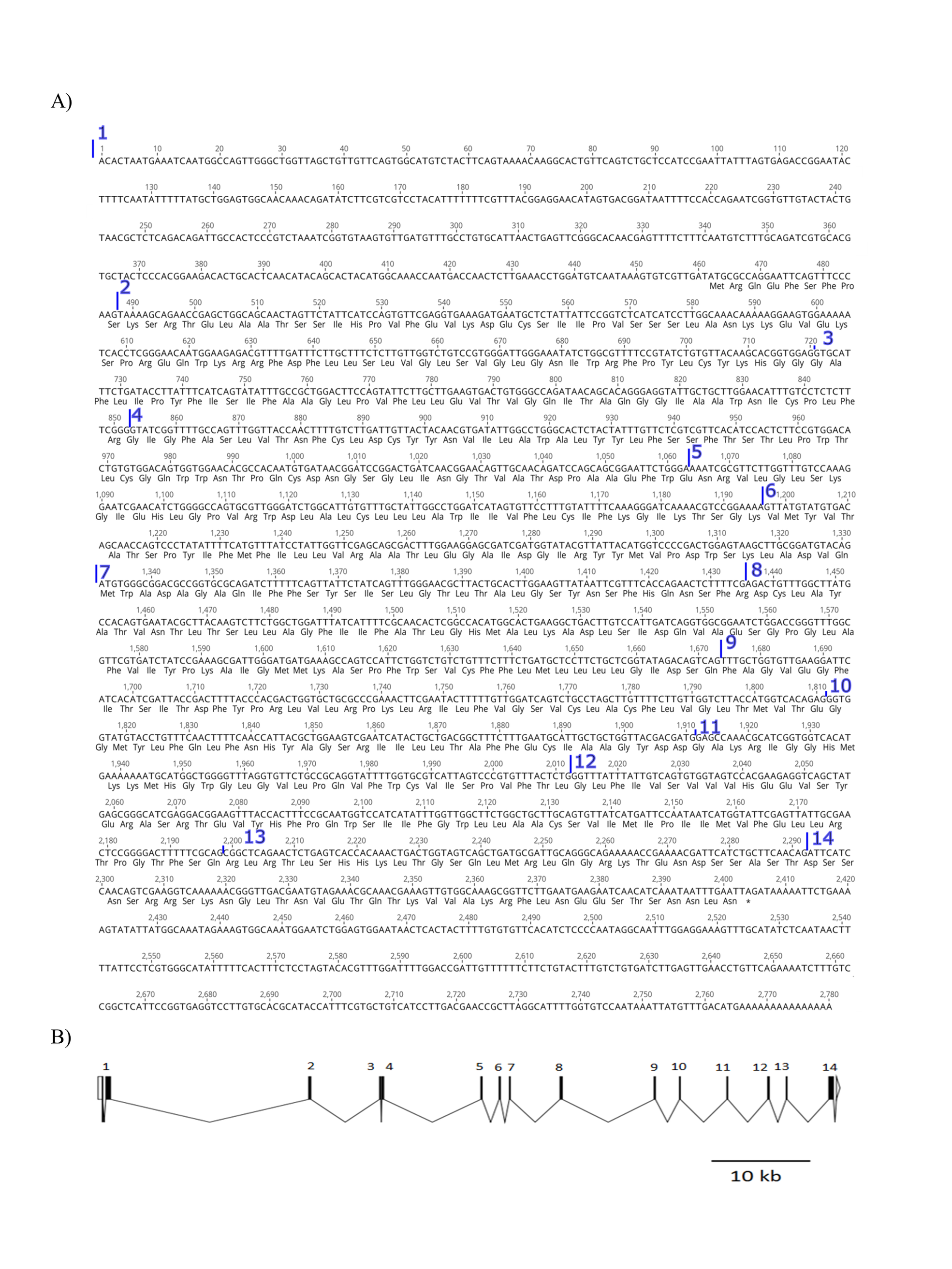

Supplement: S1 Fig — Primary structure of the F. hepatica taurine transporter (FhTauT) (A) and exon-intron structure of its gene (B). A: The cDNA of FhTauT and its conceptual translation. Amino acids are shown in three letter code. The blue lines mark the borders of consecutive exons, which are numbered (bold numbers). B: Exons and introns are indicated by boxes and lines, respectively. Coding sequences are shown as full boxes; the 5'- and 3'-untranslated regions are represented as white boxes. The analysis was performed by comparing the cDNA with the genomic sequences deposited in the worm base (http://parasite.wormbase.org/) using the Splign web tool (https://www.ncbi.nlm.nih.gov/sutils/splign/splign.cgi). The figure was generated by Exon-Intron Graphic Maker (http://www.wormweb.org/exonintron). The scale bar represents 10 kb. (TIF) [file pntd.0006428.s001.tif]

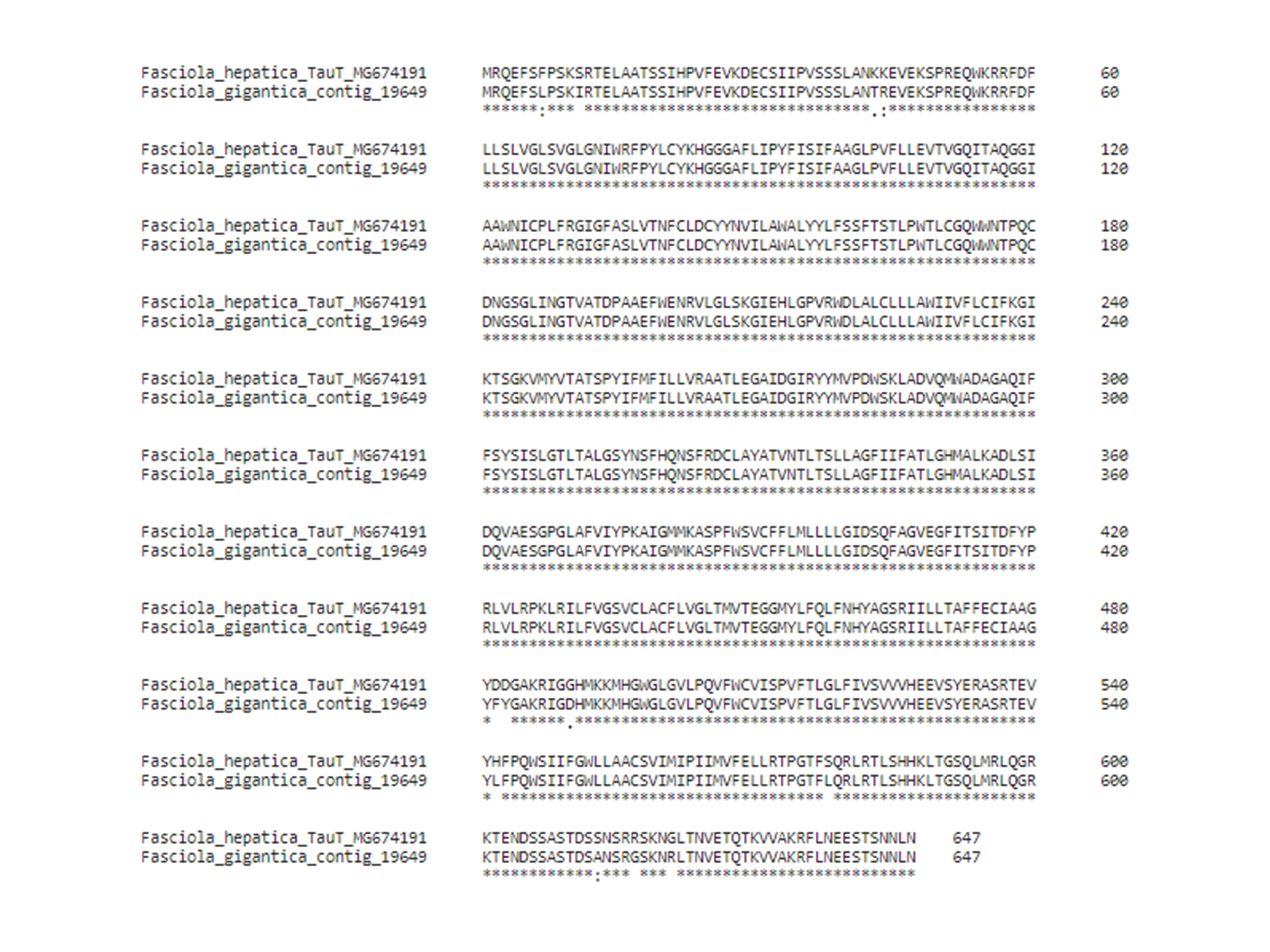

Supplement: S2 Fig — The sequence of the F. gigantica transporter was retrieved from the transcriptome deposited in (http://bioinfosecond.vet.unimelb.edu.au/index.html) [ref. 22] by BLAST search and aligned with that of the F. hepatica TauT by using Clustal Omega (version 1.2.4). The two proteins differ in 12 out of 647 positions (98% identity). Identical residues are marked by asterisks, highly (3) and less well-conserved residues (2) by colons and dots, respectively, and dissimilar residues (7) by blank spaces. The differences cluster in the N- and C-termini (i.e., before residue 57 and after residue 571); the other four changes are in intracellular loop 5 (IL5) and extracellular loop 6 (EL6). (TIF) [file pntd.0006428.s002.tif]

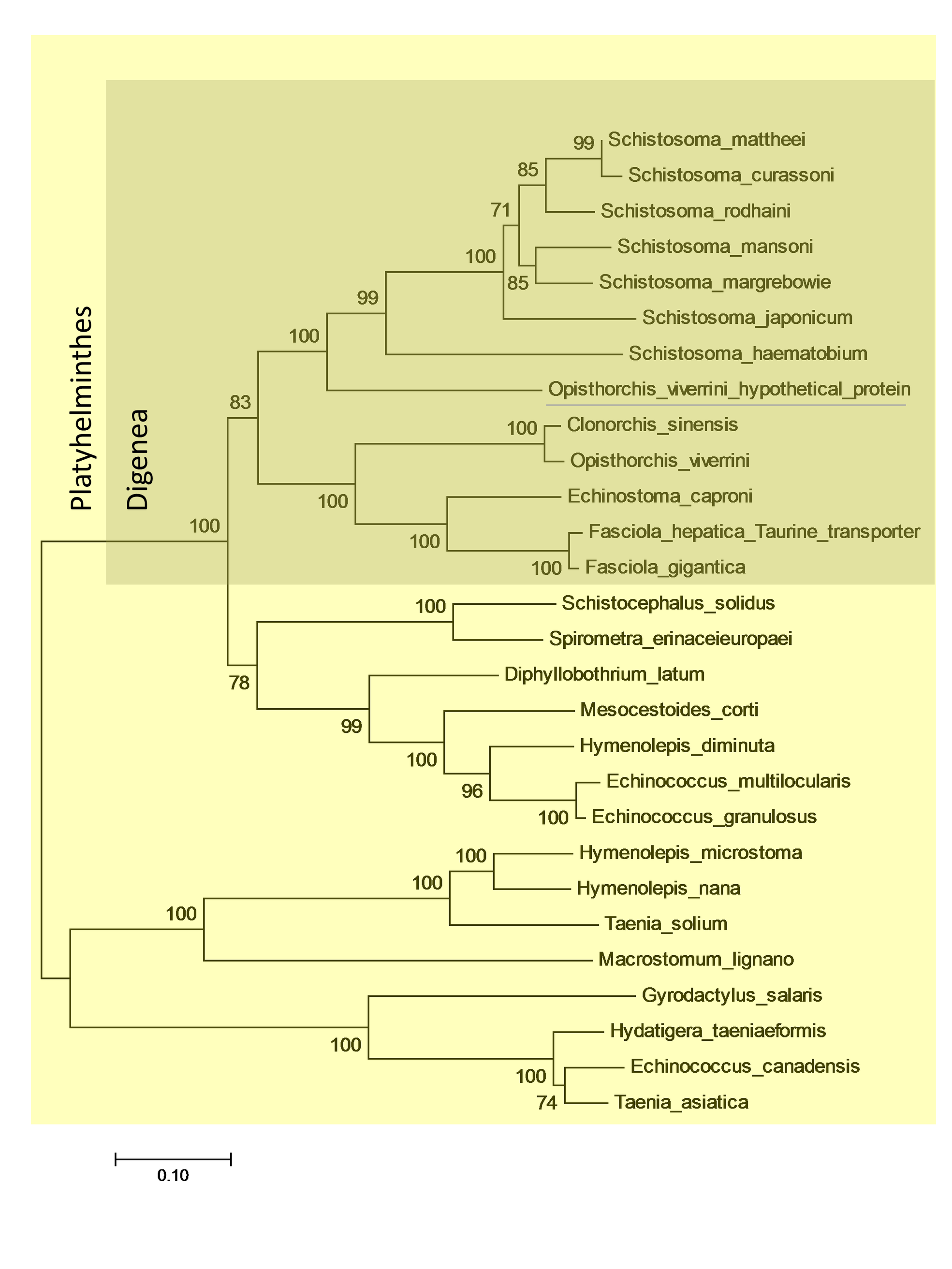

Supplement: S3 Fig — The phylogenetic tree was constructed with the neighbor-joining method implemented in MEGA 7 [34]: the amino acid sequence of FhTauT was compared to that of orthologues found in Platyhelminthes available in WormBase (for accession numbers of sequences see S1 Table.). We also found an orthologue in Protopolystoma xenopodis, Trichobilharzia regenti and in Schmidtea mediterranea, but we did not include these, because only very short sequences were available. Bootstrap valued (1000 replicates, Poisson correction) are shown next to the branches. The scale bar reflects the likelihood that a change in amino acid occurred on any given branch. Digenean transporters/contigs are highlighted in the gray area, where the underlined sequence is the one originally derived from NCBI, and used to generate the tree in Fig 2. The yellow area marks the transporters/contigs from other Platyhelminthes. (TIF) [file pntd.0006428.s003.tif]

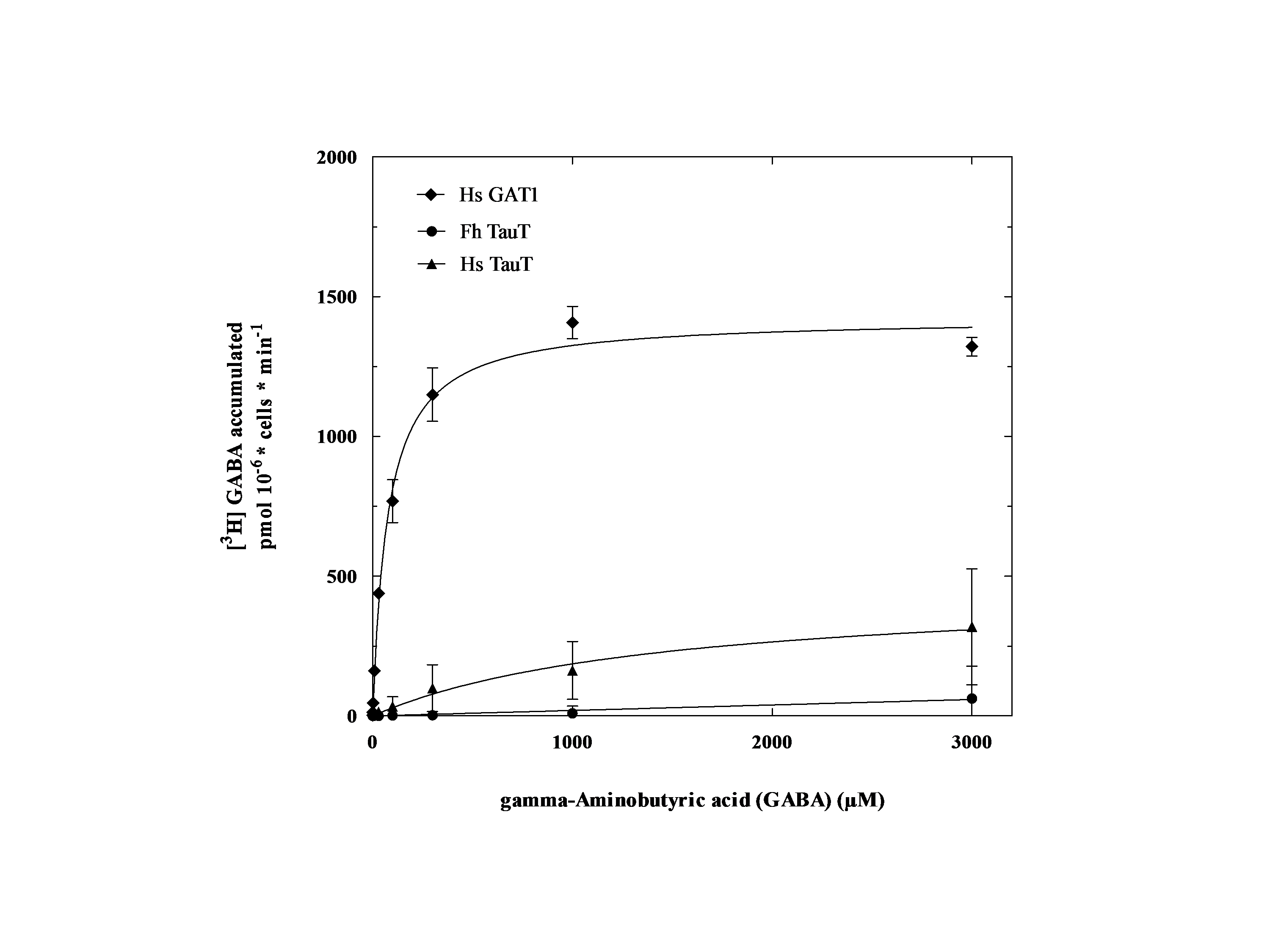

Supplement: S4 Fig — HEK293 cells (105/ well) stably expressing the human GABA-transporter-1 (HsGAT1, diamonds), the F. hepatica taurine transporter (FhTauT, circles) and the human taurine transporter (HsTauT, triangles) were incubated in the presence of the indicated concentrations of [3H]GABA. The specific activity was progressively diluted by addition of unlabeled GABA (from 9 Ci/mmol to 9 Ci/mol). After three minutes the reaction was stopped and the accumulated radioactivity was determined by liquid scintillation counting. Data are means ± S.D. of at least two independent experiments performed in triplicate. (TIF) [file pntd.0006428.s004.tif]

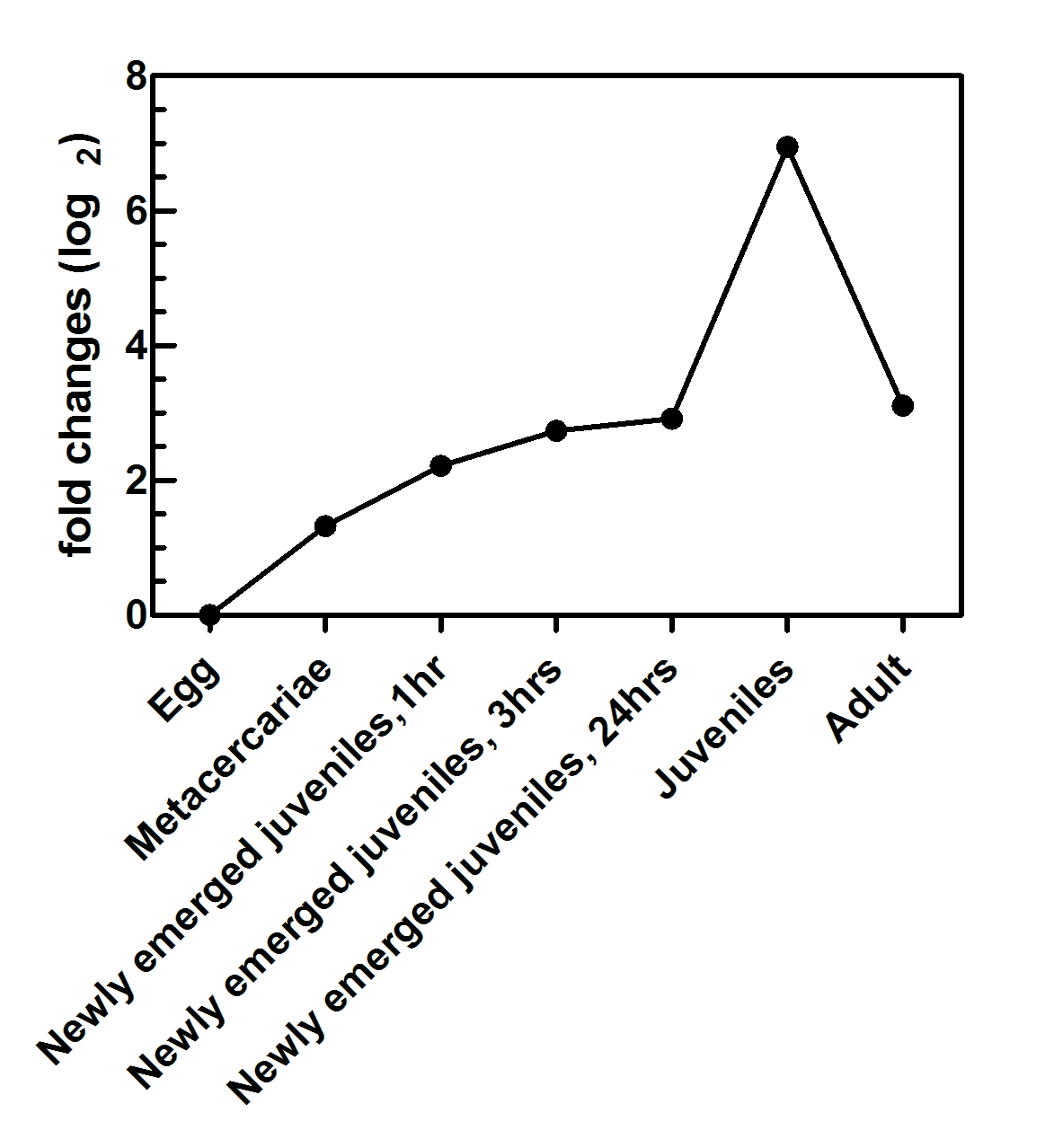

Supplement: S5 Fig — RNAseq data were extracted from the WormBase with the built-in BLAST tool (PRJEB6904). The expression level of FhTauT was quantified by the number of reads per million base pairs in different developmental stages (i.e. eggs, metacercariae, newly emerged juveniles after 1,3 and 24hrs, juveniles and adults) [66]. The reads were normalized to the expression levels in eggs (= 1) and plotted as fold-increase over this level (log2). (TIF) [file pntd.0006428.s005.tif]
